# Enhanced U-Net with Attention Mechanisms for Improved Feature Representation in Lung Nodule Segmentation

**DOI:** 10.2174/0115734056386382250902064757

**Published:** 2025-09-11

**Authors:** Thin Myat Moe Aung, Arfat Ahmad Khan

**Affiliations:** 1 College of Computing, Khon Kaen University, Khon Kaen, Thailand

**Keywords:** Pulmonary nodule segmentation, U-Net, Attention mechanisms, Deep learning, Medical image analysis, LUNA16 dataset, Computer-aided diagnosis

## Abstract

**Introduction:**

Accurate segmentation of small and irregular pulmonary nodules remains a significant challenge in lung cancer diagnosis, particularly in complex imaging backgrounds. Traditional U-Net models often struggle to capture long-range dependencies and integrate multi-scale features, limiting their effectiveness in addressing these challenges. To overcome these limitations, this study proposes an enhanced U-Net hybrid model that integrates multiple attention mechanisms to enhance feature representation and improve the precision of segmentation outcomes.

**Methods:**

The assessment of the proposed model was conducted using the LUNA16 dataset, which contains annotated CT scans of pulmonary nodules. Multiple attention mechanisms, including Spatial Attention (SA), Dilated Efficient Channel Attention (Dilated ECA), Convolutional Block Attention Module (CBAM), and Squeeze-and-Excitation (SE) Block, were integrated into a U-Net backbone. These modules were strategically combined to enhance both local and global feature representations. The model’s architecture and training procedures were designed to address the challenges of segmenting small and irregular pulmonary nodules.

**Results:**

The proposed model achieved a Dice similarity coefficient of 84.30%, significantly outperforming the baseline U-Net model. This result demonstrates improved accuracy in segmenting small and irregular pulmonary nodules.

**Discussion:**

The integration of multiple attention mechanisms significantly enhances the model’s ability to capture both local and global features, addressing key limitations of traditional U-Net architectures. SA preserves spatial features for small nodules, while Dilated ECA captures long-range dependencies. CBAM and SE further refine feature representations. Together, these modules improve segmentation performance in complex imaging backgrounds. A potential limitation is that performance may still be constrained in cases with extreme anatomical variability or low-contrast lesions, suggesting directions for future research.

**Conclusion:**

The Enhanced U-Net hybrid model outperforms the traditional U-Net, effectively addressing challenges in segmenting small and irregular pulmonary nodules within complex imaging backgrounds.

## 1 INTRODUCTION

As the most frequently diagnosed cancer, lung cancer is also the leading cause of mortality among cancer patients worldwide. According to the latest global estimates from the

**Fig. (1):**
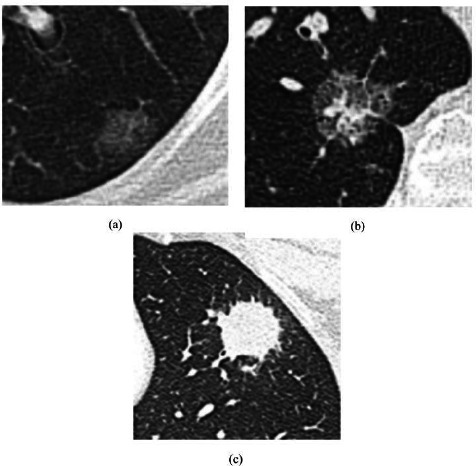
Categorization of lung nodules: (**a**) Ground-glass nodule, (**b**) Part solid nodule, (**c**) Solid nodule.Available online under the terms of the Creative Commons Attribution Non-Commercial License 3.0. [1].

International Agency for Research on Cancer (IARC), nearly 2.5 million people were diagnosed with lung cancer in 2022, with over 1.8 million deaths reported. It is widely recognized as one of the most lethal malignant tumors, posing a severe threat to human health and life. Pulmonary nodules, defined as circular or irregular lesions in the lungs measuring 3 cm or smaller, are considered precursors to lung cancer. These nodules may appear as single or multiple lesions with well-defined or blurred edges, and imaging typically reveals them as regions of increased density. Pulmonary nodules are categorized into three types based on their density: Ground-Glass Nodules (GGNs, Fig. **1a**), Part-Solid Nodules (PSNs, Fig. **1b**), and Solid Nodules (SNs, Fig. **1c**) [1]. Although it is undeniable that all lung cancers originate from pulmonary nodules, not all pulmonary nodules indicate early-stage lung cancer. This is because pulmonary nodules can be either benign or malignant, and only malignant nodules have the potential to progress into lung cancer. According to the China Anti-Cancer Association (CACA),70–80% of pulmonary nodules are benign, while the likelihood of malignancy in pulmonary nodules ranges from 20–30%. Since pulmonary nodules may serve as early indicators of lung cancer, it is crucial to stratify their risk. This enables early identification of potential lung cancers while avoiding unnecessary harm and costs associated with over-investigating low-risk nodules. Early lung nodule diagnosis and lowering cancer-related mortality have thus emerged as crucial research topics [2].

Deep learning-based Computer-Aided Diagnostic (CAD) systems have been the main driver of the impressive advancements in lung cancer diagnosis lately. These techniques not only eliminate the need for human feature engineering but also substantially enhance the efficiency and accuracy of diagnoses [3]. For instance, Convolutional Neural Networks (CNNs) can automatically recognize disease patterns and detect lesions in medical images without manual intervention. In the detection of pulmonary nodules, CNNs have proven to be particularly effective, enhancing diagnostic accuracy and efficiency while reducing false negatives and false positives. As deep learning technologies have advanced, CNNs have emerged as a key component of CAD systems and are widely used in the diagnosis and screening of conditions, including breast and lung cancer [4]. In 2015, a study suggested a Fully Convolutional Network (FCN) that incorporated upsampling layers (also called deconvolution layers) and replaced fully connected layers with convolutional layers, achieving the first pixel-level dense classification. The application of this network led to marked progress in the accuracy and efficiency of image segmentation tasks [5]. In the same year, U-Net was introduced, becoming the most widely adopted convolutional neural network architecture in medical image segmentation. By effectively capturing both contextual and local information, U-Net achieved outstanding results across various biomedical segmentation tasks [6]. Subsequently, numerous variants of U-Net have been proposed to address specific challenges. For example, U-Net++ enhances feature propagation and refinement by incorporating dense skip connections into nested U-shaped subnetworks, making it particularly suitable for tasks requiring precise boundary segmentation [7]. Similarly, Attention U-Net incorporates Attention Gates (AGs) in skip connections to dynamically adjust the importance of features across different scales, effectively suppressing irrelevant background information and emphasizing target structures. This selective feature enhancement significantly improves segmentation accuracy, especially in small-target segmentation tasks with complex backgrounds. These advancements have further optimized segmentation performance and feature extraction capabilities [8]. Following these developments, the ECANodule model was introduced, balancing performance and computational efficiency. Leveraging lightweight 1D convolution to dynamically adjust channel importance effectively enhanced feature representation and fusion capabilities, delivering excellent performance in pulmonary nodule detection [9]. However, traditional UNet and its variants still face limitations, such as difficulty in capturing long-range dependencies and effectively integrating multi-scale features. These issues become problematic when segmenting small or irregularly shaped nodules in complex anatomical contexts. The inability to capture long-range dependencies makes it difficult for traditional U-Net models to aggregate contextual information from distant regions, which is essential for distinguishing nodules from similar surrounding tissues. Furthermore, the detection of nodules with different sizes and shapes is restrained by insufficient multi-scale feature fusion, especially for small nodules that are easily missed because of the limited resolution in deeper layers. Therefore, conventional U-Net architectures often suffer from missed detections or inaccurate boundaries when dealing with nodules embedded in complex anatomical contexts or with unclear boundaries. While recent studies have introduced various methods to address these limitations, including the integration of attention mechanisms, further improvements are needed to optimize feature representation across different scales and resolutions.

Inspired by these advancements, this study proposes an enhanced U-Net model to improve the segmentation of small or irregular pulmonary nodules, particularly in complex anato-mical backgrounds. The proposed model integrates four comp-lementary attention mechanisms: SE Block, Dilated ECA, CBAM, and SA. These modules are strategically embedded within the network architecture to enhance feature represen-tation across different stages of the encoder–decoder pipeline.

Specifically, SE Blocks are applied to skip connections to strengthen multi-scale feature fusion and preserve important semantic features. The Dilated ECA module is introduced in the encoder to enhance channel-wise feature representation and expand the receptive field, allowing the model to better capture long-range contextual information. CBAM is integrated into the decoder to refine spatial and channel-wise attention during feature reconstruction, improving localization precision. Additionally, the SA module is incorporated in the early stages of the encoder to retain spatial details that are critical for detecting small or irregular nodules. This coordinated attention design enables the model to balance global context with local detail, resulting in more accurate and robust segmentation performance.

To sum up, the main contributions of this study are as follows:

1. An enhanced U-Net architecture is proposed, integrating four complementary attention mechanisms: SA, Dilated ECA, CBAM, and SE Block. Each module is designed to target a specific aspect of feature learning. Through this hybrid combination, both spatial and channel attention mechanisms are leveraged to improve multi-scale representation and long-range dependency modeling.

2. A task-specific attention placement strategy is introduced: SE Blocks are applied to skip connections to enhance multi-scale feature fusion; CBAM and Dilated ECA are embedded in the decoder and encoder, respectively, to refine semantic features and model global context; and the SA module is incorporated in the early encoding stages to retain spatial details critical for segmenting small and irregular nodules.

3. A Particle Swarm Optimization (PSO) based framework is developed to tune key loss function hyperparameters, including the BCE weight, Dice weight, and weight decay. This approach contributes to improved training stability and better segmentation performance.

4. Extensive experiments are conducted on the LUNA16 dataset, and it is demonstrated that the proposed model consistently outperforms the baseline U-Net.

The remainder of this paper is organized as follows. Section 2 reviews the traditional U-Net, attention mechanisms, and their variants. Section 3 introduces the proposed methodology in detail. Section 4 describes the dataset and experimental setup. Section 5 presents the experimental results and related discussions. Finally, it concludes the study and outlines potential directions for future work.

## 2 RELATED WORKS

### 2.1 U-Net

A traditional medical image segmentation architecture called U-Net was put forth in 2015. U-Net consists of an encoder and a decoder; the encoder gradually lowers the input image's resolution in order to extract features, while the decoder gradually raises the resolution intended to produce segmentation results. Furthermore, it has skip connections, which allow both high-level and low-level feature information to be used to increase segmentation accuracy and robustness. There are two primary ways that U-Net differs from FCN. First, the U-Net can use high-level features and spatial information from the encoder for more precise segmentation because of its totally symmetric structure. In contrast, the decoder structure of FCN is relatively simple, employing only a single deconvolution operation without subsequent convolutional layers. The second difference lies in the skip connections: FCN uses addition operations, whereas U-Net adopts concatenation. U-Net's encoder-decoder design is a distinguishing characteristic, often employed in applications such as image compression and noise reduction. This design has proven to be highly effective and straightforward when applied to image segmentation. Initially developed for biomedical image segmentation, the U-Net is now widely used across various image-processing tasks.

### 2.2 U-Net Variants for Volumetric Medical Imaging

U-Net has been an impact success, but its two-dimensional variants face some difficulties when applied to volumetric data. Specifically, there is a lack of ability to capture three-dimensional spatial context information. Extended versions, such as 3D U-Net [10], have been proposed to address this challenge. This model processes medical volume data more effectively by transitioning from 2D operations to 3D convolutions, max pooling, and up-convolutions, enabling better spatial context capture across three dimensions. As an infrastructure, U-Net has spawned numerous extensions and improvements, enabling multiple evolutions, including 3D U-Net and models incorporating attention mechanisms. Accordingly, U-Net is now a crucial component of medical image segmentation.

### 2.3 Emergence of Attention Mechanisms in CNNs

For image processing applications, Convolutional Neural Networks (CNNs) have emerged as the standard in the fields of deep learning and computer vision. However, further enhancing neural network performance and efficiency has become a crucial problem due to the growing size of datasets and model complexity. To address this issue, a study proposed the SE Network [11], which introduces the channel attention mechanism as an effective solution. Traditional methods have primarily focused on enhancing CNNs by improving feature encoding capabilities in the spatial domain. In contrast, SE Network introduces the SE Block, which focuses on channel-wise feature recalibration. By dynamically learning the importance of each channel, SE Block assigns adaptive weights to individual channels, enabling the network to amplify informative feature maps and suppress less relevant ones. The core idea of SE Block is to capture global information of each channel through Global Average Pooling (GAP), followed by generating channel-wise weights using two Fully Connected (FC) layers. The learnt weights are then used in the recalibration procedure to modify the channel-wise feature responses. Through the use of the loss function, this adaptive learning occurs during network training, enabling the model to highlight useful features and enhance performance. Notably, the SE Block is not a stand-alone network architecture but rather a plug-and-play submodule that can be incorporated into existing classification or detection models. Although using SE Blocks inevitably results in more parameters and higher processing costs, the performance gains are often substantial, especially for applications like object detection and image classification.

The success of SE Block has sparked significant interest in attention mechanisms that aim to enhance feature representation by dynamically focusing on important information. A study proposed the CBAM [12], which also extends the idea of channel attention by introducing spatial attention. To improve feature refinement, CBAM sequentially integrates Channel Attention and Spatial Attention, in contrast to SE Block, which only concentrates on channel-wise recalibration. Cross-channel and spatial information are inevitably mixed in convolution operations. The channel axis, or “what” to focus on, and the spatial axis, or “where” to focus on, are the two primary dimensions that CBAM highlights significant elements across. By dynamically recalibrating the relevance of specific channels and spatial positions within the feature map, CBAM enables the model to prioritize crucial information, ultimately enhancing its overall performance.

In a similar vein, a study presented the Efficient Channel Attention (ECA) module [13], a novel channel attention mechanism designed to enhance the performance of deep Convolutional Neural Networks (CNNs) with the fewest additional parameters and computing costs. Whereas the SE Block uses two Fully Connected (FC) layers to generate channel weights, ECA uses a fast one-dimensional convolution of size k, where k is adaptively determined as a function of the channel dimension C. ECA's attention computation serves as a natural regularization method, requiring no additional regularization techniques and being less prone to overfitting. Its strong adaptability allows it to be easily integrated into various convolutional neural networks without extensive modifications or adjustments. Building upon this, a study presented the Dilated ECA module [14], which additionally integrates dilated convolutions to enhance channel attention efficiency and concurrently record local and global contextual information. The Dilated ECA module effectively models multi-scale features, making it particularly well-suited for tasks requiring fine-grained segmentation of small targets or irregular structures.

### 2.4 Advancements in U-Net Variants with Attention Mechanisms

In recent years, numerous studies have focused on enhancing the U-Net and its derivatives by integrating advanced attention mechanisms. For example, ECA U-Net [15] integrates the Efficient Channel Attention (ECA) module, enabling channel interaction without dimensionality reduction while dynamically adjusting channel weights to emphasize critical features. The ECA module adaptively selects kernel sizes based on network depth by employing a one-dimensional convolution kernel for channel interaction, thereby striking an effective balance between performance and computational efficiency. Furthermore, the decoder, designed with deconvolution, effectively restores feature map resolution. Additionally, ResDSda_U-Net [16] also combines advanced attention mechanisms and multi-scale feature extraction modules to improve segmentation performance. The model integrates SimAM, Channel Spatial Attention (CSA), and Cross Channel Attention (CCA) modules, which work together to enhance the representation of not only channel but also spatial features. ResDSda_U-Net is ideally suited for segmenting intricate and small structures, like lung nodules, as the Dense Atrous Spatial Pyramid Pooling (DASPP) module efficiently captures multi-scale features.

Thereafter, a study with an improved U-Net framework was proposed [17], incorporating a residual expansion structure (Res-Dil) and a channel-space joint attention mechanism. The residual expansion structure leverages multi-scale convolutions to capture rich contextual information, while the attention mechanism dynamically focuses on key feature regions. Likewise, MANet [18] introduces a multi-branch attention-assisted learning mechanism, combining multi-task learning with enhanced attention-based designs. To make pulmonary nodule feature depiction better, the model, which is based on the U-Net architecture, includes the Projection Module, Fast Cascading Context Module, and Boundary Enhancement Module.

Furthermore, a study proposed a novel network architecture, TPFRNet [19], which combines a Transformer pooling module and a dual attention feature recombination module. The model utilizes the Transformer pooling module to effectively extract global contextual information while incorporating a multi-scale convolution module to capture local features.

These surveyed models demonstrate the effectiveness of incorporating advanced attention mechanisms and multi-scale processing into the U-Net framework, significantly improving feature extraction for medical image segmentation. However, research on hybrid designs that comprehensively address long-range dependencies and complex segmentation tasks, particularly for small or irregular structures, remains limited. To bridge this gap, our study proposes an Enhanced U-Net model that integrates multiple attention mechanisms to improve feature representation across different scales and resolutions, thereby enhancing segmentation performance in such scenarios. Based on recent studies, various attention-based U-Net variants have been developed to enhance segmentation accuracy and contextual understanding. For instance, ECA-UNet applies channel-wise attention using efficient convolution, while ResDSda-U-Net integrates multiple attention modules, such as SimAM and CCA/CSA, to improve feature representation, which may increase architectural complexity. In contrast, our approach seeks to maintain a balance between model expressiveness and computational cost. U-Det [20], on the other hand, adopts a lightweight structure without attention mechanisms. In contrast, our method combines SE, CBAM, and Dilated ECA modules to jointly enhance local detail extraction, spatial awareness, and multi-scale contextual encoding. While not necessarily more complex, this design emphasizes complementary feature processing strategies.

## 3 MATERIALS AND METHODS

### 3.1 Model Architecture

Multiple attention mechanisms are integrated into our proposed model, which is an improved U-Net design, including the SA Module, Dilated ECA Module, SE Block, and CBAM. The model uses a three-layer encoder-decoder structure, as illustrated in Fig. (**2**). While the decoder uses upsampling blocks and skip connections to restore spatial resolution, the encoder uses downsampling blocks with attention modules to gradually extract multi-scale information.

Specifically, the encoder begins with an initial convolutional block, followed by three downsampling stages. Each stage includes a DoubleConv3D block, which consists of two successive 3 × 3 × 3 convolutional layers, each followed by Batch Normalization and ReLU activation, and includes a residual connection to enhance gradient flow. In particular, the first two stages integrate convolutional layers with the SA module in order to emphasize small structures and retain their features in deeper layers. By contrast, the third stage combines a convolutional layer with the Dilated ECA module, which focuses on enhancing global representation by capturing multi-scale context and refining channel-wise feature importance.

Unlike the CBAM conception, which prioritizes channel attention followed by spatial attention, our model adopts a reverse strategy. It first focuses on spatial attention in the shallow layers and then shifts to channel attention in the deeper layers. In the shallow layers, the model primarily processes low-level features, such as textures, edges, and small targets. The SA module helps the model identify important spatial locations (*e.g*., lung nodule regions), ensuring that these critical details are preserved and not diluted by subsequent convolutional operations. On the other hand, as the number of channels increases in the deeper layers, the feature map resolution falls. At this stage, the model emphasizes extracting semantic information and understanding global contextual relationships. To enhance the model's overall efficiency, the channel attention module is better suited for capturing global dependencies and focusing on key semantic features. In medical imaging, the spatial location of small targets like lung nodules is particularly crucial, making our model’s design more effective for retaining spatial details in the shallow layers, whereas CBAM is more suitable for high-level tasks with richer semantic features.

On the other hand, the decoder consists of three upsampling blocks that mirror the encoder structure. The first and third upsampling blocks integrate CBAM to further refine spatial and channel-wise features, ensuring effective feature reconstruction. Furthermore, SE blocks are applied to the first and last skip connections to pass only the most relevant information, thereby improving the precision of feature reconstruction and segmentation results. Table **1** shows the model's comprehensive overview.

In summary, the proposed design leverages SA modules in shallow layers to enhance local feature extraction and Dilated ECA in deeper layers for robust global feature integration. Besides, CBAM in the decoder ensures that refined encoder features are effectively reconstructed, while SE blocks in skip connections further highlight critical features. This architecture is deliberately designed to progressively enhance spatial detail retention and global context modeling through stage-specific attention placement, enabling precise segmentation of small, complex structures such as lung nodules. Finally, the model generates the segmentation mask through a 1 × 1 convolution.

### 3.2 Spatial Attention Module

The CBAM, which combines channel and spatial attention mechanisms to improve feature representation, is the model from which the SA module employed in this study was modified. To overcome specific feature extraction problems, our suggested model employs the SA module independently in the encoder, while CBAM uses SA as part of its sequential attention process. In the encoder, the SA module is independently incorporated into the first and second downsampling stages. This design ensures the retention of critical spatial features, particularly those corresponding to small and irregular structures such as lung nodules, which are crucial for accurate segmentation. By focusing on spatially significant regions, the SA module effectively highlights key details such as edges and shapes while suppressing irrelevant background noise.

Consequently, this improves overall segmentation accuracy as well as the robustness of the model. Moreover, the independent application of the SA module allows the model to prioritize spatial information without being influenced by channel recalibration, ensuring more precise feature extraction in the early stages.

Using max pooling and average pooling operations along the channel dimension, the SA module aggregates the input feature map X to create a spatial attention map, as shown in Fig. (**3**). This process results in two feature maps, *F_avg_* and *F_max_*, both with dimensions of (1, *D*, *H*, *W*) as defined by the following Eqs. (**1** and **2**).

**Table:** 

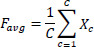	(1)

**Table:** 

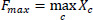	(2)

Where *C* denotes the total count of channels, the input’s *C^th^* channel of the feature map is indicated by *X_C_*, and *c* is the index of a specific channel, ranging from 1 to *C*. Following this, these features are concatenated along the channel dimension, yielding *F_cat_*, with the dimensions of (2, *D*, *H*, *W*), the operation depicted as Eq. (**3**).

**Table:** 

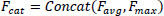	(3)

Where the concatenation operation across the channel dimension is indicated by *Concat*. Then the concatenated feature map *F_cat_* is convolved with a kernel size *k* = 7, and a sigmoid function is applied. This operation generates a single-channel feature map *M_SA_*, which represents the importance weights of each spatial location with dimensions (1, *D*, *H*, *W*) as described by the following Eq. (**4**).

**Table:** 

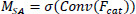	(4)

Where σ is the sigmoid function. Finally, the spatial attention map is element-wise multiplied with the input feature map X to produce the weighted output feature map F_out. The shape of this output feature map is identical to that of the input feature map. The operation is depicted below Eq. (**5**) as follows:

**Table:** 

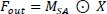	(5)

Where 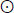 denotes element-wise multiplication. Overall, the SA module plays a pivotal role in balancing local detail retention and global spatial awareness, ensuring precise feature representation in medical image analysis.

### 3.3 Dilated Efficient Channel Attention

Tasks involving image processing and recognition heavily depend on global information. By capturing the overall structure and macro-level features of an image, global information significantly improves the accuracy of model classification and recognition [21]. This capability is particularly important in tasks that require a comprehensive understanding of semantic content. While local information is indispensable for handling fine-grained details, global information provides the broader contextual background needed to interpret these details effectively. For instance, in a basketball scene, the position of the ball alone may not reveal the player's action. However, by combining global information, such as the court and the audience, it can be inferred that the player is shooting the ball.

Combining global and local information enhances the model's overall imaging comprehension and task performance. Global information offers macro-level context, while local information focuses on detailed features like textures and edges. Their integration not only facilitates precise boundary localization in segmentation tasks but also ensures overall consistency, leading to significant improvements in segmentation quality.

In the third layer of the proposed encoder, the primary focus is on capturing global contextual relationships rather than merely emphasizing localized spatial features. This is particularly crucial for understanding the relationship between the lesion and its surrounding environment, especially for small or irregular structures such as lung nodules. Through max pooling and average pooling procedures on the input feature map, SA adds a certain amount of global context, but its ability to represent global information remains limited. These pooling operations perform only simple statistical aggregations along the channel dimension, failing to capture complex cross-regional dependencies within the image. As a result, while SA provides some global information, it is insufficient for effectively modeling long-range dependencies, which are critical for deeper layers of the encoder.

We therefore offer the Dilated ECA module to the encoder's third layer in order to close this gap. The Dilated ECA module captures long-range dependencies while preserving computational speed, which is specifically intended to improve global contextual awareness. Unlike pooling-based operations, Dilated ECA leverages dilated convolutions and efficient channel attention mechanisms to comprehensively model the inter-channel relationships and spatial dependencies within the feature map.

Initially, the input feature map (*C*, *D*, *H*, *W*) is split into four sub-feature maps as (*C/*4, *D*, *H*, *W*) along the channel dimensions. Each sub-feature map is processed using depth-wise separable convolution with different dilation rates (*d* = 1, 2, 5, 7) as depicted in Fig. (**4**). This operation generates four output feature maps (*X*_1_, *X_2_*, *X*_3_, *X*_4_,), as Eqs. (**6** to **9**) below:

**Table:** 

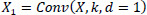	(6)

**Table:** 

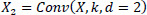	(7)

**Table:** 

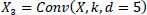	(8)

**Table:** 

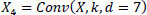	(9)

Here, *X* stands for the input sub-feature map, *k* is the kernel size of the convolution operation, which is dynamically chosen using an adaptive mapping function *ѱ*(*C*) based on the channel dimension *C*. Unlike the original paper, which uses fixed kernel sizes for convolution operations, this dynamic adjustment of *k* ensures that the receptive field is proportional to the input channel dimensions, effectively balancing computational efficiency with coverage of local and global dependencies, and *d i*s the dilation rate. Dilation rates of 1 and 2 capture fine-grained local details, while rates of 5 and 7 focus on global contextual information. These convolutions effectively extract multi-scale features, enhancing the representation of both local and global context. The dynamic kernel size *k* is computed as the following Eq. (**10**).

**Table:** 

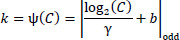	(10)

Where *C* is the channel dimension, γ and *b* are hyperparameters controlling the mapping, and |*t*|*_odd_* denotes the nearest odd integer of *t*. In this implementation, γ and *b* are set to 2 and 1, respectively.

The resulting feature maps are then concatenated to form a new multi-scale feature map, *X_concat_*, which combines the outputs of the convolutions with different dilation rates as the following Eq. (**11**).

**Table:** 

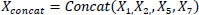	(11)

Where *Concat* represents the concatenation process across the channel dimension.

After obtaining the multi-scale feature map, Global Average Pooling (GAP) is applied to compress the spatial dimensions, producing a channel-wise global descriptor. The output feature map of the shape (*C*, 1, 1, 1, 1), where C is the number of channels, is the result of the GAP operation aggregating the feature map along its spatial dimensions. The GAP operation is expressed in Eq. (**12**) below:

**Table:** 

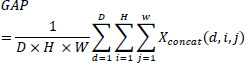	(12)

Subsequently, a 1D convolution with a dynamic kernel size *k* = *ѱ*(*C*) is applied to perform local cross-channel interaction. Then, the output is passed through a ReLU activation function to introduce nonlinearity. Afterward, the feature weights are normalized into the range (0, 1), using a Sigmoid function, producing channel-wise attention weights *F_out_* by Eq. (**13**).

**Table:** 

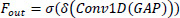	(13)

Where *δ* denotes the ReLU activation function and *σ* is the Sigmoid function.

Finally, the attention weights are applied to the original concatenated feature map *X_concat_* through element-wise multiplication, resulting in the final output feature map as given in Eq. (**14**).

**Table:** 

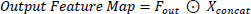	(14)

Where 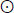 denotes element-wise multiplication.

### 3.4 Convolutional Block Attention Module

In our model, the CBAM is integrated into the first and third layers of the decoder to enhance feature reconstruction by refining both spatial and channel-wise attention. Using CBAM in the decoder strengthens the model’s ability to accurately reconstruct segmentation masks from encoded features. CBAM selectively focuses on important channels and spatial locations, improving feature refinement and ensuring precise segmentation boundaries. In segmentation tasks, where obtaining good results requires capturing fine spatial features and global context, this is especially beneficial.

During upsampling, CBAM in the decoder's first layer preserves shallow feature details, thereby avoiding the loss of important spatial information like boundaries and patterns. In the final layer, it emphasizes global contextual understanding and strengthens semantic feature reconstruction, ensuring the segmentation mask is both precise and reliable.

In contrast, the second layer of the decoder serves as a transition phase, where feature information is relatively balanced between shallow and deep layers. At this stage, the focus is on efficient feature propagation without additional attention mechanisms to avoid computational redundancy. Including CBAM here could lead to overuse of attention modules and unnecessary computational overhead.

Incorporating CBAM in the decoder complements the attention mechanisms used in the encoder, ensuring a balanced and comprehensive attention strategy across the model. While encoder attention mechanisms focus on extracting and refining global and local features, CBAM ensures that these refined features are effectively reconstructed into the final segmentation mask. By leveraging spatial attention to recover lost details and channel attention to prioritize key features, CBAM significantly enhances the accuracy and reliability of segmentation.

This module combines Channel Attention that highlights important channel features and Spatial Attention mechanisms that focus on significant spatial locations to sequentially refine the input feature map, as shown in Fig. (**5**).

The Channel Attention mechanism is shown in the upper portion of the diagram, which is indicated by the blue box. First, the input feature *X* is subjected to average pooling and maximum pooling. Across the spatial dimensions (*D*, *H*, *W*), generating two-channel descriptors *F_avg_* and *F_max_* with the shape (*C*, 1, 1, 1). Subsequently, they undergo processing via a shared Multi-Layer Perceptron (MLP), which comprises two completely connected layers. To generate a channel attention map *M_c_*, ReLU is applied after the first layer and Sigmoid after the second, as given in Eq. (**15**).

**Table:** 

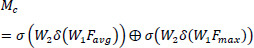	(15)

Where *σ* denotes the Sigmoid activation function, δ represents the ReLU activation function, and *W*_1_ and *W*_2_ are the learned weights of the MLP. Correspondingly, the element-wise addition denoted by 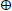, which aggregates the outputs from the two branches of the MLP after applying the ReLU and Sigmoid activation functions to each channel descriptor.

This concludes the Channel Attention mechanism. The attention map *M_c_*, generated from the Channel Attention process, is applied to the original input feature *X via* element-wise multiplication, selectively enhancing or suppressing channel-specific information as described in Eq. (**16**).

**Table:** 

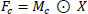	(16)

Where 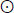 denotes element-wise multiplication.

The resulting output *F_c_* retains the same shape as the input feature map, which is (*C*, *D, H, W*), and serves as the input for the Spatial Attention module, highlighted in the green box. The module begins by applying average pooling and max pooling operations along the channel dimension to the input *F_c_*. These pooling operations generate two spatial descriptors, *F_avg_* and *F_max_*, both of which retain the spatial dimensions (*D*, *H*, *W*) resulting in the shape of (1, *D*, *H*, *W*). Note that *F_avg_* and *F_max_* here are different from the descriptors used in the Channel Attention module, as they are computed along the channel dimension rather than the spatial dimension. A combined spatial description with the shape (2, *D*, *H*, *W*) is then created by concatenating these descriptors along the channel dimension.

Subsequently, the combined spatial descriptor is passed through a 3D convolutional layer with a kernel size of 7 × 7 × 7. This operation captures spatial dependencies across the depth, height, and width dimensions. After that, Eq. (**17**) processes the output through a Sigmoid activation function to produce the spatial attention map *M_s_*, which has the shape (1, *D*, *H*, *W*).

**Table:** 

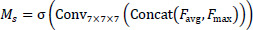	(17)

Where the pooling procedures' outcomes are *F_avg_* and *F_max_, Concat* denotes the concatenation operation along the channel dimension, and the Sigmoid activation function is marked as *σ*.

After generating the spatial attention map *M_s_*, it is applied to the input feature map *F_c_ via* element-wise multiplication, resulting in the final refined feature map *F_s._* This operation emphasizes important spatial regions while maintaining the original spatial dimensions. The resulting feature map *F_s_* retains the same shape as *F_c_* by the following Eq. (**18**).

**Table:** 

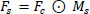	(18)

Where 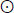 denotes element-wise multiplication and *F_s_* is the output feature map.

### 3.5 Squeeze and Excitation Block

With regard to improving the representation of significant channels, the SE Block's fundamental concept involves Global Average Pooling (GAP) to capture each channel's global properties. Then, the relationships between channels are learned using fully connected layers and activation functions, allowing the network to recalibrate channel-wise feature responses and prioritize the most informative ones, as illustrated in Fig. (**6**).

In our model, the SE Block is incorporated into the skip connections, specifically in the first and third layers connecting the encoder and decoder. The first layer benefits from enhancing shallow, detailed features, ensuring that low-level spatial information is preserved, while the third layer strengthens the semantic and global features, improving the decoder's capacity to reconstruct high-level representations. The second layer, as a transitional phase with balanced feature complexity, is excluded to avoid redundancy and minimize computational overhead. In skip connections, the primary goal is to transfer features from the encoder to the decoder, providing critical information to guide the reconstruction of the output. These features already contain a mixture of local and spatial details from the encoder. By introducing the SE Block, the model enhances the decoder's ability to focus on the most significant features while suppressing distractions from less relevant information.

The SE Block emphasizes channel-wise importance, ensuring that key features are not overshadowed during the reconstruction process. This selective attention helps maintain the integrity of the segmentation output and significantly enhances the overall effectiveness of the decoder.

It starts by applying Global Average Pooling (GAP) to the input feature map *F* to capture global information across all spatial dimensions. This operation reduces the spatial dimensions (*D*, *H*, *W*) into a single scalar value for each channel, producing a channel-wise global descriptor. After that, it is processed through two Fully Connected (FC) layers. In correspondence to successfully learn the relevance of each channel, the first FC layer uses a ReLU activation function to improve the representation, while the second FC layer uses a Sigmoid activation function to compute normalized channel-wise attention weights *S* by the following Eq. (**19**).

**Table:** 

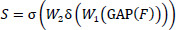	(19)

Where *σ* is the Sigmoid activation function, *δ* represents the ReLU activation function, and *W*_1_ and *W*_2_ are the learned weights.

Finally, the computed channel-wise attention weights *S* are applied to the original input feature map *F via* element-wise multiplication as Eq. (**20**), resulting in the output feature map *F_output_*.

**Table:** 

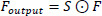	(20)

Where 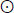 stands for element-wise multiplication and *F_output_* is the output feature map.

### 3.6 Loss Function

Medical image segmentation tasks present several difficulties, especially when dealing with small and irregular objects like lung nodules. These challenges include inherent class imbalance between target and background regions and the need for precise boundary delineation. A composite loss function, incorporating Intersection over Union (IoU) Loss, Dice Loss, and Binary Cross-Entropy (BCE) Loss, is brought up to effectively overcome these challenges. Each component targets a specific aspect of segmentation: BCE Loss [6] ensures pixel-level classification accuracy, Dice Loss [22] improves segmentation performance for target regions, particularly for imbalanced datasets, and IoU Loss [23] ensures that the predicted results align more closely with the overall shape and boundaries of the target area. The total loss function is defined by the following Eq. (**21**).

**Table:** 

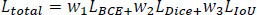	(21)

Where *w*_1_, *w*_2_, *w*_3_ represent the weights for each component.

BCE Loss acts as a pixel-wise classification loss, optimizing the predicted probability for each pixel to match the ground truth by the following Eq. (**22**).

**Table:** 

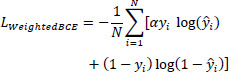	(22)

Here, *ŷ_i_* indicates the expected probability following Sigmoid activation, and *y_i_* is the ground truth label for the *i^th^* pixil. The parameter *α* represents the weight assigned to positive samples. To address the class imbalance issue, *α* is used to give higher importance to the underrepresented target regions, ensuring that the loss function emphasizes these areas during optimization.

Dice Loss maximizes the overlap between the predicted and ground truth regions. By emphasizing accurate segmentation of small target regions, it is particularly effective in handling class imbalance. The Dice Loss is defined by the following Eq. (**23**).

**Table:** 

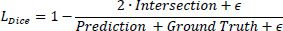	(23)

Where, *Intersection* is the overlapping region between the predicted and ground truth regions, the *Prediction* represents the total number of pixels in the predicted region, the *Ground Truth* denotes the total number of pixels in the ground truth region and *ϵ* refers to the small smoothing factor that is added to avoid division by zero.

By taking into account both overlapping and non-overlapping regions, the Intersection over Union (IoU) loss assesses the global alignment between the anticipated and ground-truth regions. The definition of the IoU loss is given in Eq. (**24**).

**Table:** 

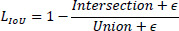	(24)

Where, *Intersection* refers to the area where the prediction and ground truth masks overlap, and the *ϵ* represents the small smoothing factor added to avoid division by zero. While *Union* indicates the entire area, including both overlapping and non-overlapping areas, that is covered by either the ground truth region or the anticipated region. The Union is calculated as the following Eq. (**25**).

**Table:** 

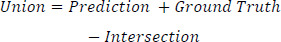	(25)

This formula ensures that the total area accounts for all parts of the prediction and ground-truth regions while avoiding double-counting of the overlapping areas.

### 3.7 Optimal Hyperparameter Selection for Loss Weights and Regularization

In this context, it is crucial to carefully select hyperparameters, such as loss weights and regularization parameters, to construct an optimal network. Therefore, we adopted Particle Swarm Optimization (PSO) [24] to efficiently determine the best combination of these hyperparameters.

Using a swarm of particles for discovering the search space, the PSO algorithm iteratively modifies the weight decay, BCE weight, and dice weight. Each particle represents a potential solution and is guided by both its own best position and the best position found by the entire swarm.

The objective function used for optimization is the validation Dice score, ensuring that the selected hyperparameters maximize segmentation performance. To ensure fairness and robustness, the optimization process was conducted on the LUNA16 dataset using consistent experimental settings. The search space for hyperparameters was constrained to maintain the sum of weights, preventing overfitting to any single component. The final optimized hyperparameters were then used to train the model. Algorithm **1** describes the flow in the PSO-based optimization process.

## 4 EXPERIMENTAL DATA

### 4.1 Datasets and Preprocessing

The experimental data used in this study is derived from the LUNA16 dataset, a challenge competition dataset extracted from the LIDC-IDRI dataset. The LUNA16 dataset is accessible to researchers upon registration via the official website: https://luna16.grand-challenge.org. The LUNA16 dataset retains only nodules with a diameter greater than 3 mm, which were annotated as nodules by at least three radiologists. It contains 888 CT scans and 1,186 positive lung nodules. Additionally, the dataset provides masks for lung parenchyma segmentation, pixel coordinates of the nodules, and the diameter of each nodule. However, since generating ground truth requires detailed contour information of the nodules, which is not included in the LUNA16 dataset, we utilized the XML files from the LIDC-IDRI dataset. These XML files contain detailed nodule contour information for each slice, which allows us to obtain the necessary annotations for further processing.

**Algorithm 1:** PSO for optimizing loss weights and weight decay.

**Inputs:**
- Dataset: Training and validation set from LUNA16
- Number of particles: *n_pop_*
- Maximum iterations: *max*_*iter*
- Inertia weight: *w*
- Cognitive and social coefficients: *c*_1_, *c*_2_
- The range for weight decay: [*w_min_*, *w_max_*]
- The range for BCE weight: [*b_min_*, *b_max_*]
- The range for Dice weight: [*d_min_*, *d_max_*]
**Outputs:**
- Optimized weight decay: *wdopt*
- Optimized loss weights: *bce*_*weight_opt_, dice_weight_opt_*
Step 1: Initialize Particles
a) Set the global best Dice score *g_best_* to zero.
b) Initialize *n_pop_* particles with random positions and velocities.
• For each particle:
• Randomly select *weight*_*decay*, *bce_weight*, *dice*_*weight* within their respective ranges.
• Calculate *iou_weight* = *max*(0.0, 1.0-*bce*_*weight*) to ensure weights sum to 1.
c) Save the initial position and velocity for each particle.
Step 2: Define Fitness Function
a) Assess the fitness of a particle's parameters *weight*_*decay*, *bce_weight*, *dice*_*weight* by:
• Training the model on the training set.
• Validating the model on the validation set.
• Compute the negative validation Dice score.
Step 3: Perform Iterations
a) While *t* < *max*_iter:
For each particle:
• Evaluate the fitness of the current position using the fitness function.
• Update the particle's personal best *p_best_* if the current fitness is better.
• Update the global best *g_best_* if the current fitness is better than the global best.
• Adjust velocity:
• Incorporate the particle's personal best *p_best_*.
• Incorporate global best *g_best._*
• Update position based on the new velocity:
• Ensure the new position stays within the defined search space.
b) Increment t.
Step 4: Build the Model with Optimized Parameters
a) Use *wd_opt_*, *bce*_*weight_opt_*, *dice*_*weight_opt_* to initialize the model.
b) Train the model with these optimized parameters.

As illustrated in Fig. (**7**), the preprocessing workflow began with lung region extraction, where the original CT images were multiplied by binary lung masks to isolate the lung parenchyma, effectively removing irrelevant background information. After this, to guarantee consistency across all samples, the extracted lung region's pixel intensity values were then normalized, followed by mean subtraction to smooth intensity variations and eliminate inherent biases in the CT images. The lung region was then resampled to a standardized voxel spacing, ensuring consistent spatial resolution for 3D analysis and model training. Subsequently, Regions of Interest (ROIs) were extracted based on nodule annotations using the center coordinates and diameter, with a focus on nodule-containing regions to enable detailed segmentation and reduce computational overhead. Finally, the preprocessed ROIs were input into the segmentation model to generate pixel-wise nodule segmentation predictions.

To ensure fair evaluation and reproducibility, we followed the official LUNA16 dataset subset division, using subsets 0–7, eight subsets, for training and subsets 8–9, two subsets, for validation. This partitioning strategy ensures that CT scans from different patients are not mixed across the training and validation sets, thereby preventing data leakage.

In total, the training set contains 963 3D patches, and the validation set contains 223. No separate test set was used in this phase; all results reported in this study are based on validation performance. The partitioning details are summarized in Table **2**.

To further clarify the dataset characteristics, we analyzed the distribution of nodule sizes in the LUNA16 dataset. As shown in Fig. (**8**), small nodules with diameters ≤10 mm account for more than 80% of all annotated nodules, indicating a highly skewed size distribution. Although nodules of varying sizes exist, our study primarily targets the segmentation of small nodules, which pose greater challenges due to their subtle appearance and irregular morphology.

To investigate class imbalance at the voxel level, we examined how training samples are constructed and analyzed the distribution of positive (nodule) and negative (background) voxels. As illustrated in Fig. (**9**), each input sample is a 3D patch of size 16 × 96 × 96, where only a few voxels are labeled as nodules, while the vast majority represent background tissue. Quantitatively, Fig. (**10**) shows that positive voxels comprise less than 1% of the total voxel count in both the training and validation sets.

To mitigate this extreme imbalance, we applied a weighted loss function by increasing the contribution of positive voxels during training. Specifically, a positive class weight of 1.56 was used, which was empirically determined to balance performance and stability. This strategy enhances the model’s sensitivity to detecting small and low-contrast nodules, improving segmentation performance under imbalanced conditions.

### 4.2 Evaluation Metrics.

The effectiveness of our model is assessed in this study using three commonly used assessment metrics: Dice Coefficient (DSC), Sensitivity (SEN), and Positive Predictive Value (PPV). The equations for these metrics are presented from Eqs. (**26** to **28**) as follows:

**Table:** 

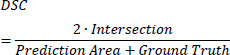	(26)

**Table:** 

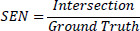	(27)

**Table:** 

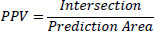	(28)

Where, the percentage of correctly predicted positive regions out of all anticipated positive regions is determined by *PPV*, *SEN* analyzes the model's ability to accurately identify the real positive regions, and *DSC* calculates the similarity between the predicted and ground truth regions.

### 4.3 Implementation Details

The experiments in this paper were conducted using Python 3.10.12, PyTorch 2.5.1, and CUDA 12.2 on a single NVIDIA L4 GPU provided by the Google Cloud Platform. The model was trained for 200 epochs with a batch size of 4, and validation was performed after each epoch. The best-performing model was selected based on the highest validation Dice score. The initial learning rate was set to 0.001 and decayed linearly throughout the training process. The AdamW optimizer was employed, and the optimal weight decay was determined through Particle Swarm Optimization (PSO), resulting in a value of 5.17 × 10^−4^. The combined loss function integrates Binary Cross-Entropy (BCE), Dice Loss, and Intersection over Union (IoU) Loss, with weights also optimized via PSO. The final loss weights were 0.2093 for BCE, 0.4702 for Dice, and 0.3205 for IoU, and a positive voxel weight α = 1.56 was applied to the BCE term. To enhance robustness and prevent overfitting, data augmentation techniques, including geometric transformations (random flip and rotation) and intensity augmentation (Gaussian noise), were applied.

### 4.4 Comparative Analysis of Optimizers

In this study, we evaluated three different optimizers—Adam, AdamW, and Radam—to identify the most suitable option for our segmentation task. After thorough comparisons, AdamW was chosen for use as it performed better in terms of computing efficiency, generalization, and convergence speed.

Introduced in 2014, Adam [25] gained significant attention for its ability to combine the advantages of momentum and RMSProp, which allowed it to adjust learning rates for each parameter adaptively. Despite its early success, Adam exhibited performance instability in certain scenarios, prompting researchers to explore alternatives, such as Stochastic Gradient Descent (SGD) with momentum. To address these challenges, AdamW [26] was proposed in late 2017, introducing a corrected implementation of weight decay. This correction improved the optimizer's regularization capabilities, enabling faster convergence and generalization. AdamW has been widely adopted in diverse applications, including computer vision and natural language processing tasks. RAdam [27], short for Rectified Adam, further builds on Adam by dynamically adjusting the adaptive learning rate based on variance. This eliminates the need for manual learning rate warm-up, making it a robust alternative in tasks with limited data or inconsistent gradients.

As summarized in Table **3**, AdamW consistently outperformed both Adam and RAdam in terms of Dice and F1 Scores, while also achieving lower average training times per epoch. When comparing the performance of optimizers, AdamW demonstrated clear advantages over both Adam and RAdam in terms of Dice score and training efficiency.

Specifically, AdamW achieved a 4.65% increase in Dice score compared with Adam, improving from 0.8056 to 0.8430, while also significantly reducing the average training time per epoch by 30.19%, from 95.45 seconds to 66.65 seconds. Similarly, AdamW outperformed RAdam with a 4.53% improvement in Dice score, rising from 0.8065 to 0.8430. However, RAdam exhibited slightly faster training, with an average epoch time of 64.77 seconds, which was marginally lower than AdamW's 66.65 seconds, reflecting a 2.91% decrease. These results highlight AdamW's ability to balance higher accuracy with competitive training efficiency, making it the optimal choice for our segmentation task.

## 5 RESULTS AND DISCUSSION

### 5.1 Comparative Performance Analysis of the Proposed and Traditional U-Net

In this section, we compare the proposed model with the traditional U-Net in terms of segmentation performance on the LUNA16 dataset. Metrics including Dice, Sensitivity (SEN), and Positive Predictive Value (PPV) were used to evaluate the segmentation accuracy. For a fair assessment, the comparison was carried out with the identical experimental setup and dataset (LUNA16).

As illustrated in Fig. (**11**), the proposed model outperformed the traditional U-Net across all metrics, achieving a higher Dice coefficient, sensitivity, and PPV. Specifically, it achieved a Dice score of 84.30%, PPV of 85.50%, and sensitivity of 83.32%, while the traditional U-Net recorded a Dice of 78.47%, sensitivity of 77.19%, and PPV of 81.05%. This improvement highlights the effectiveness of the proposed model in capturing better contextual features and providing more robust predictions. Besides, the balanced improvement in sensitivity and PPV indicates that the proposed model not only detects the majority of nodules accurately but also minimizes false positives. This balance is particularly crucial in medical image segmentation tasks, where both false positives and false negatives can have significant clinical implications.

### 5.2 Ablation Studies

To further investigate the effectiveness of each attention mechanism in the proposed model, we conducted an ablation study by incrementally adding the following modules: SA, Dilated ECA, and CBAM, followed by the SE block in the full model. The segmentation results are visually compared in Fig. (**12**), based on three representative slices selected from the LUNA16 dataset validation set. These slices were chosen to reflect a range of nodule characteristics, including variations in size, shape complexity, and contrast, providing a comprehensive view of the model’s behavior across different scenarios.

As shown in the figure, each row corresponds to a representative slice selected from the validation set, and each column demonstrates the output under a different model configuration. Specifically, Fig. (**12a**) displays the original CT input slice, while Fig. (**12b**) presents the corresponding ground truth mask. Fig. (**12c**) shows the prediction generated by the baseline U-Net. Fig. (**12d**) displays the output of the UNet enhanced with SA (UNet+SA). Fig. (**12e**) shows the prediction after integrating Dilated ECA alongside SA (UNet+SA+Dilated ECA). Fig. (**12f**) illustrates the result of further incorporating the CBAM (UNet+SA+Dilated ECA+CBAM). Finally, Fig. (**12g**) demonstrates the segmentation produced by the full model, which includes an additional block (UNet+SA+Dilated ECA+CBAM+SE).

**Fig. (12):**
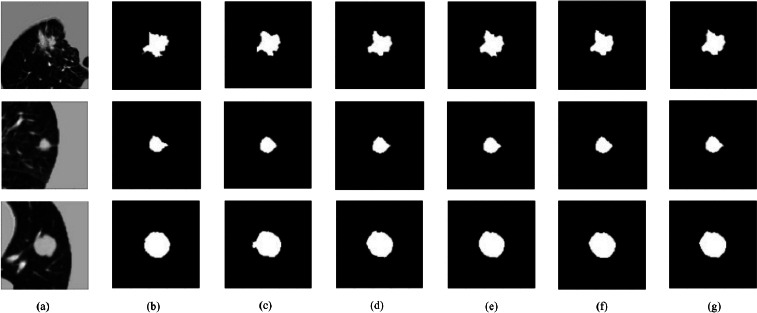
Visual comparison of segmentation outputs on three representative slices from the LUNA16 dataset validation set. Each row corresponds to a distinct input slice, while columns (a)–(g) represent: (**a**) original input slice, (**b**) ground truth mask, (**c**) baseline UNet, (**d**) UNet + SA, (**e**) UNet + SA + Dilated ECA, (**f**) UNet + SA + Dilated ECA + CBAM, and (**g**) the full proposed model UNet + SA + Dilated ECA + CBAM+ SE.

In the first row, which corresponds to a representative validation slice, the baseline UNet, Fig. (**12c**), captures the overall nodule shape, but some boundary regions are truncated, and the structure appears slightly fragmented with blurry connections. When SA is introduced (Fig. **12d**), the segmentation improves notably around the top and bottom edges, producing a denser and more cohesive mask. However, minor blocky artifacts are still present in the center. With the addition of the Dilated ECA module (Fig. **12e**), the predicted region becomes fuller and more consistent. The top boundary slightly expands outward, suggesting enhanced channel-level feature capture, although slight over-segmentation occurs. Incorporating CBAM (Fig. **12f**) further sharpens the edges and smooths the contour. The model becomes more responsive to local textures, enabling it to distinguish finer boundary details. Finally, the full model (Fig. **12g**) yields the most accurate result. The segmentation closely overlaps with the ground truth, exhibiting smooth boundaries and strong internal coherence. Even minor artifacts, such as the top-left spiculation, are significantly reduced, indicating better shape continuity and noise suppression.

In the second row of the figure, which presents a nodule with subtle protrusions and relatively low contrast, the baseline UNet (Fig. **12c**) generates a roughly circular segmentation mask centered at the correct location. However, the predicted boundaries are noticeably smaller than the ground truth, failing to capture outward extensions of the nodule, resulting in under-segmentation. With the addition of the SA module (Fig. **12d**), the boundary becomes smoother, and the overall contour better resembles the ground truth. Nevertheless, the prediction still lacks sufficient coverage, especially in the outermost regions. Once the Dilated ECA module is incorporated (Fig. **12e**), the segmentation noticeably improves and the boundaries expand outward, closely matching the true nodule shape. The combination of spatial and channel attention enhances the model’s understanding of complex structures, although minor over-segmentation is observed at the bottom edge. When integrating CBAM in Fig. (**12d**), the mask aligns more naturally with the nodule's contour, and local boundary details are refined. The model captures subtle spatial cues without significant false positives. Finally, the full model (Fig. **12g**) delivers the most accurate segmentation. It achieves near-perfect alignment with the ground truth, maintaining a smooth and artifact-free contour. This result demonstrates the effectiveness of deeper feature integration and decoder-level refinements in the proposed architecture.

In the third row, the selected slice presents a well-defined nodule with smooth boundaries and high contrast against the background tissue. The baseline U-Net (Fig. **12c**) successfully captures the overall shape and location of the nodule, but the output mask shows a slightly irregular boundary and a small protrusion on the left side, indicating some minor over-segmentation artifacts. With the inclusion of the SA module (Fig. **12d**), the predicted contour becomes more rounded and compact, with better alignment to the ground truth. This improvement highlights the SA module's ability to enhance focus on salient regions with strong contrast. However, small boundary inaccuracies remain in some areas. The addition of the Dilated ECA module (Fig. **12e**) significantly improves segmentation performance, particularly along the lower right boundary. The result appears more compact and robust to background interference, suggesting that Dilated ECA's multi-scale channel attention effectively complements SA in capturing finer structural details. When CBAM is incorporated (Fig. **12f**), the mask remains generally consistent but displays slight pixel-level instability near the boundary. While CBAM helps enhance the network’s attention to local texture features, it also introduces mild oversensitivity to background noise, leading to minor false positives. Finally, the output of the full model (Fig. **12g**), which includes the SE block, demonstrates a highly consistent and accurate segmentation. The predicted mask closely aligns with the ground truth, showing smooth and continuous contours with minimal segmentation errors. These results suggest that the combination of multiple attention mechanisms contributes to improved boundary delineation and overall segmentation quality.

### 5.3 Performance Comparison with Other Segmentation Models Using the LUNA16 Dataset

This section compares the proposed model with eight other segmentation models, U-INET, HAU-Net, SSCV-Net, SKV-Net, CSE-GAN U-Net, U-Det, and MANet, to assess its performance in more detail, using the LUNA16 dataset. U-INT [28] introduces a resource-efficient design with bidirectional feature networks and the Mish activation function to enhance segmentation. HAU-Net [29], a hierarchical attention U-Net, incorporates attention mechanisms in its decoder to better handle the heterogeneity and small sizes of lung nodules. SSCV-Net [30], built on an improved V-Net architecture, uses short skip connections to enhance feature propagation and aggregation. SKV-Net [31] integrates selective kernel blocks into V-Net to improve multi-scale feature extraction. In CSE-GAN U-Net [32], a discriminator with spatial squeeze-and-channel excitation modules is paired with a U-Net-based generator featuring simultaneous SE blocks to enhance feature recalibration. U-DET, the Bi-FPN-based encoder-decoder model, employs a weighted binary cross-entropy loss, a Bidirectional Feature Pyramid Network for multi-scale feature fusion, and the Mish activation function for regularization, aiming to rectify the class inequality and enhance accuracy for small nodules. In the end, MANet, which is based on a U-Net backbone, presents a multi-branch attention auxiliary learning mechanism with three novel modules: the Boundary Enhancement Module, the Fast-Cascading Context Module, and the Projection Module. These modules boost segmentation performance by enhancing both local and global context features. The goal of this comparison analysis is to demonstrate the benefits of the proposed model with regard to robustness and segmentation accuracy.

As shown in Fig. (**13**) and Table **4**), the proposed model consistently outperforms other segmentation methods across key evaluation metrics on the LUNA16 dataset. Specifically, Fig. (**13a**) illustrates the Dice Coefficient (DSC) comparison, Fig. (**13b**) shows the Positive Predictive Value (PPV), and Fig. (**13c**) presents the Sensitivity (SEN) across all models. The proposed model achieves the highest Dice coefficient (84.30%) and Positive Predictive Value (PPV) (85.50%) among all compared models, indicating superior segmentation accuracy and precision. Specifically, it surpasses MANet (83.61%) and HAU-Net (83.34%) by 0.69% and 0.96%, respectively, and significantly outperforms SSCV-Net (69.10%) by a margin of 22.0%.

While U-Det demonstrates the highest sensitivity (92.24%), its relatively lower PPV (78.92%) suggests a trade-off between recall and precision. In contrast, the proposed model maintains a more balanced performance, achieving competitive sensitivity (83.32%) while retaining the best precision. This balance is particularly crucial in medical image segmentation, where both false positives and false negatives carry significant clinical implications.

Overall, these results highlight the robustness and generalization capability of the proposed method in handling complex segmentation challenges such as small nodules, noisy boundaries, and heterogeneous lung structures, making it a promising solution for practical deployment in pulmonary nodule analysis.

### 5.4 Training Performance and Results Analysis

This section presents the training dynamics and segmentation performance of the proposed model. As shown in Fig. (**14**), the Dice score curves over 200 epochs demonstrate a stable and effective learning process. The training curve increases rapidly during the initial epochs and gradually reaches a plateau, indicating convergence. In parallel, the validation curve closely follows the training trend, suggesting strong generalization ability and minimal overfitting. Both curves stabilize at high Dice scores, highlighting the robustness and consistency of the model across different samples.

To qualitatively evaluate segmentation performance, Fig. (**15**) presents the results of six representative validation cases. Each row includes the original CT slice, the ground truth mask, and predictions from multiple segmentation models, arranged from top to bottom as follows: UNet, UINet, HAUNet, SSCV-Net, SKV-Net, CSE-GAN UNet, U-Det, MANet, and the proposed method. Each column represents a distinct case selected to illustrate common challenges in lung nodule segmentation, such as low contrast, small size, irregular boundaries, and complex background textures.

In the first column, the target nodule is located in a low-contrast region with limited intensity differences from surrounding tissues. Most models, including U-Net, UINET, and SKV-Net, tend to produce slightly enlarged segmentation masks, leading to mild over-segmentation. While SSCV-Net and U-Det achieve relatively accurate localization, they still exhibit minor boundary deviations. In contrast, the proposed model generates a compact, well-aligned mask that aligns more closely with the ground truth. This case highlights the challenge of segmenting nodules within complex backgrounds. The proposed model demonstrates improved boundary adherence and reduced background leakage, contributing to enhanced specificity under low-contrast conditions.

In the second case, the target nodule is very small and positioned at the upper left edge of the lung, a challenging scenario due to its size and peripheral position. Many models struggle with this case, such as U-Net and SKV-Net; they fail to detect the nodule entirely, producing empty predictions. In contrast, UINet detects a partial mask close to the correct location, but the output is fragmented and not tightly aligned with the ground truth. Meanwhile, SSCV-Net and MANet successfully identify the target region but tend to produce slightly over-segmented masks, resulting in enlarged or distorted shapes compared to the ground truth. Notably, U-Det provides a compact and well-localized mask, although it slightly under-segments the target. By comparison, HAUNet and CSE-GAN UNet capture the correct location but produce slightly enlarged masks with mild over-segmentation. The proposed model accurately identifies the location and generates a coherent mask with a more consistent contour. Although the segmentation is marginally enlarged, it maintains better shape integrity. This case shows the difficulty of segmenting small, peripherally located nodules. The proposed model shows relatively better sensitivity and structural precision, making it more robust in difficult edge cases.

In the third case, the target nodule presents a moderately challenging scenario which is relatively small, centrally located within a homogeneous lung region, and lacking strong contrast differentiation. While all benchmarked models successfully detect the nodule, their segmentation performance reveals notable variations in precision. Models like U-Net, UINet and MANet produce reasonably good segmentations but tend to slightly overestimate the boundary, whereas SKV-Net shows the opposite tendency, producing undersized segmentations. On the other hand, models such as U-Det and CSE-GAN UNet generate predictions that closely resemble the ground truth in both shape and position with minimal deviation. The proposed model generates one of the most accurate and visually consistent results, with a smooth boundary and a mask that closely matches the ground truth in both size and shape. This case illustrates the importance of boundary precision when segmenting nodules with regular morphology. The proposed model demonstrates a relatively strong ability to preserve shape fidelity while minimizing over- or under-segmentation, which can be beneficial for clinical interpretation.

The fourth column illustrates a mid-sized nodule with irregular boundaries embedded in a complex lung background. While most models successfully identify the nodule, their ability to preserve boundary details varies significantly. Specifically, UNet, UINet, and SK-Net produce relatively smooth masks, yet miss some fine-grained contour transitions. In contrast, HAUNet and SSCV-Net slightly overestimate the boundary, whereas MANet captures the general shape well but tends to smooth out boundary irregularities. On the other hand, CSE-GAN UNet and U-Det offer better shape approximation along with improved contour fidelity. Notably, the proposed model produces the most faithful representation of the nodule’s shape, not only capturing its irregular structure but also maintaining accurate alignment with the ground truth. This case illustrates the importance of capturing morphological complexity, especially in nodules with non-uniform edges. As evidenced by the results, the proposed model demonstrates a superior ability in retaining structural variations, delivering more anatomically faithful segmentations.

In the fifth column, the nodule is small and located near the lung boundary, surrounded by a noisy background, which increases the difficulty of precise segmentation. Although all benchmarked models successfully detect the nodule, their predictions differ in terms of shape regularity and boundary control. Both UNet and SKV-Net successfully identify the nodule region, with UNet producing a slightly enlarged and smoother mask, while SKV-Net shows a closer approximation in size but with less precise edge definition. UINet, HAUNet, and SSCV-Net yield reasonably accurate predictions but lose some boundary detail through excessive smoothing. In contrast, CSE-GAN UNet and MANet show a tendency to over-segment the nodule, producing slightly oversized masks with more rounded or extended boundaries. U-Det provides better alignment with the ground truth. The proposed model produces the most balanced result, achieving a good compromise between size accuracy and boundary detail preservation. This case highlights the importance of robustness when segmenting small nodules near the boundary in noisy environments.

The sixth case is a particularly complex case involving a large, irregular nodule with blurred boundaries and lobulated shape, presenting significant segmentation challenges. UNet under-segments the nodule, producing a noticeably smaller and overly smooth mask that omits key features, particularly the upper-right protrusion. In contrast, UINet improves the boundary alignment with the ground truth but still fails to preserve the finer details. Meanwhile, HAUNet captures the overall shape accurately but tends to slightly expand the mask along the edges, and the upper boundary shifts inward. SSCV-Net also preserves the general structure but introduces minor distortion, and some peripheral branches disappear. In contrast, SKV-Net produces a more compact shape with simplified and angular edges, leading to a less anatomically accurate representation. Although MANet successfully captures the overall shape of the nodule, it tends to over-smooth the boundary, leading to the loss of critical features, such as small bifurcations and subtle notches. This indicates a reduced sensitivity to local structural complexity. The proposed model achieves the most anatomically faithful segmentation. It preserves both the global shape and local boundary variations, maintaining sharp, well-aligned contours with minimal distortion. The resulting mask exhibits high consistency with the ground truth in terms of shape and area, demonstrating strong capability in handling irregular and detail-rich nodule structures.

Overall, the proposed method exhibits consistently strong segmentation performance across various challenging scenarios, including low-contrast nodules, small targets, irregular shapes, and complex backgrounds. While not always producing the best result in every case, it tends to achieve more reliable boundary delineation, good shape retention, and reduced over- or under-segmentation errors compared to many existing methods. These advantages are largely attributed to the integration of attention mechanisms and improvements in both the encoder and decoder, which together enable a balanced representation of local details and global context.

To verify the statistical significance of the observed performance improvement, we conducted a paired t-test between the Dice scores of the proposed model and the baseline UNet across multiple runs. The paired t-test yielded a p-value of 8 × 10^−6^ and a t-statistic of 29.36, indicating a statistically significant improvement (p < 0.05) in Dice performance. The average Dice score improvement was 0.0500, with a 95% confidence interval of [0.0453, 0.0547], confirming that the enhancement is consistent and not due to random chance.

The results presented above suggest that the proposed model demonstrates competitive segmentation performance on the LUNA16 dataset, with improved boundary coherence and reduced false positives compared with baseline methods. A higher Dice score indicates greater overlap between the predicted mask and the ground truth, reflecting better segmentation precision—an essential factor for accurate lesion delineation in clinical practice. Additionally, the model achieves a relatively high sensitivity, which reduces the risk of missed nodules and is especially important for early-stage disease detection. Its highest PPV among the compared methods implies fewer false positives, thereby helping to reduce diagnostic errors and unnecessary follow-up procedures. While the model does not achieve the highest sensitivity, it maintains a clinically meaningful balance across key metrics. This balance between sensitivity and precision is particularly crucial in medical applications, where both false negatives and false positives can lead to serious consequences. Such robustness is especially valuable in segmenting small or irregular nodules under low-contrast or noisy imaging conditions.

However, several limitations should be acknowledged. The evaluation was conducted only on the LUNA16 dataset, which, despite its popularity, includes nodules from a limited number of individuals and lacks annotations for more complex or overlapping cases. These constraints may limit the model’s generalizability to broader clinical settings. In addition, its performance under varying image noise levels and across different CT scanners remains untested. Future research could focus on multi-center validation, domain adaptation, and robustness analysis to further enhance the clinical applicability of the proposed approach.

## CONCLUSION AND FUTURE WORK

We introduced a hybrid model in this research that combines multiple attention mechanisms to improve feature representation and gather more detailed contextual data. The Dilated ECA module improves channel-wise feature interaction, enabling the capture of global context, while the problem of long-range reliance in conventional U-Net models is partially addressed by CBAM, which combines channel and spatial attention to concentrate on important regions and reduce irrelevant background. To tackle the difficulty of distinguishing target regions from complex backgrounds, SA is applied in the early network stages to capture spatial relationships, aiding in the separation of targets and backgrounds. Additionally, CBAM and SE Block provide fine-grained feature selection, which reduces background interference and improves robustness in complex environments. The segmentation of small or irregular nodules is a recognized challenge, which we addressed through the use of Dilated ECA for multi-scale feature extraction, contributing to improved localization of small nodules. SE Block further improves multi-scale feature fusion in skip connections, ensuring that small nodule features are preserved during the decoding stage. The SA module also aids in capturing spatial relationships of small targets, improving segmentation accuracy. Through the synergistic effects of these modules, the proposed model demonstrates an enhanced ability to segment small and irregular nodules in challenging scenarios. Results from experiments on the LUNA16 dataset confirm the model's efficacy, showing significant gains in segmentation accuracy over the baseline U-Net.

Despite advancements, certain limitations remain. The LUNA16 dataset, which is well-known yet has constraints, was used to verify the proposed model. Due to its small size and the fact that it only includes nodules from a small number of individuals, the dataset might not accurately represent the variety and complexity of actual clinical situations. Additionally, it lacks annotations for more challenging cases, such as overlapping or extremely small nodules, which limits its applicability in more diverse clinical settings. Furthermore, while the proposed algorithm demonstrates promising performance, there remains potential for refinement, particularly in optimizing computational efficiency and further improving segmentation accuracy in challenging cases.

To increase the model's robustness across a range of clinical situations, future studies may validate it using larger and more diverse datasets. Exploring other medical imaging datasets, such as datasets for liver lesions or brain tumors, could further demonstrate the model's adaptability and generalization capabilities. Incorporating multi-modal data, such as PET-CT or MRI, or extending the approach to handle more complex nodule characteristics, including overlapping and multi-nodule cases, represents a promising direction for enhancing the model's clinical utility.

## Figures and Tables

**Fig. (2):**
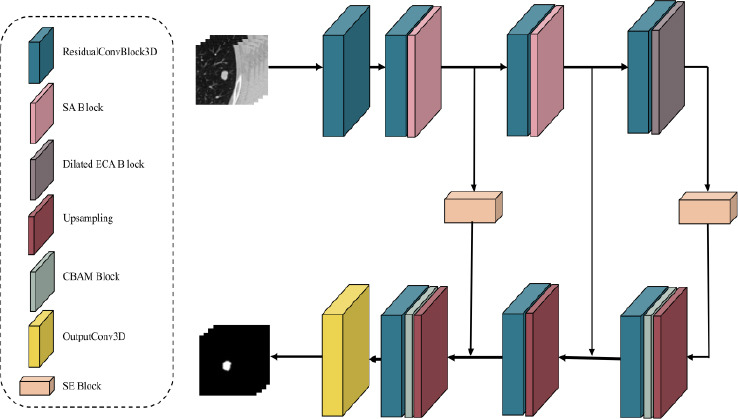
The composition of the model.

**Fig. (3):**
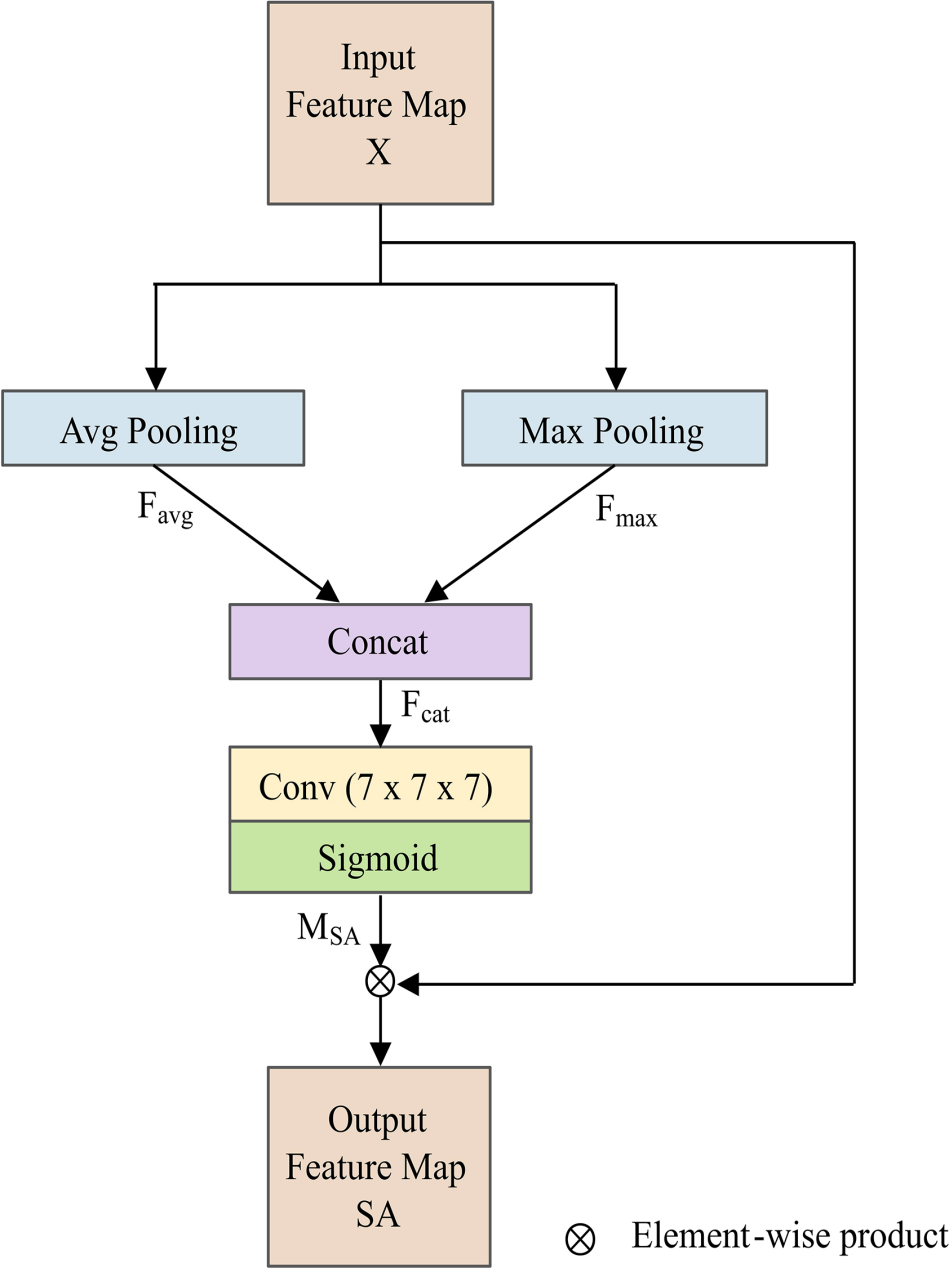
Spatial attention block.

**Fig. (4):**
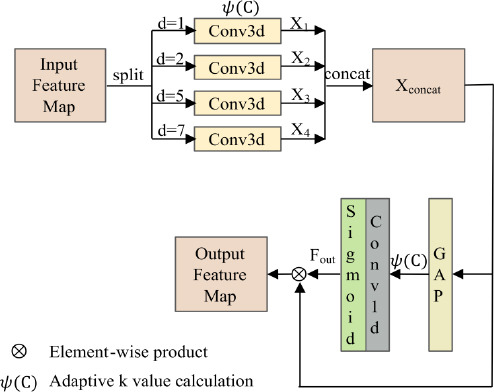
Dilated efficient channel attention block.

**Fig. (5):**
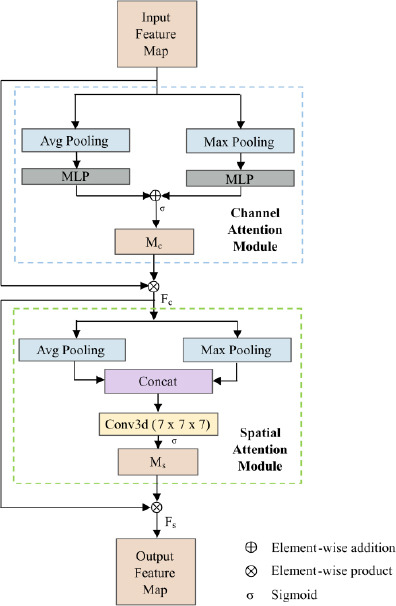
Convolutional block attention module.

**Fig. (6):**
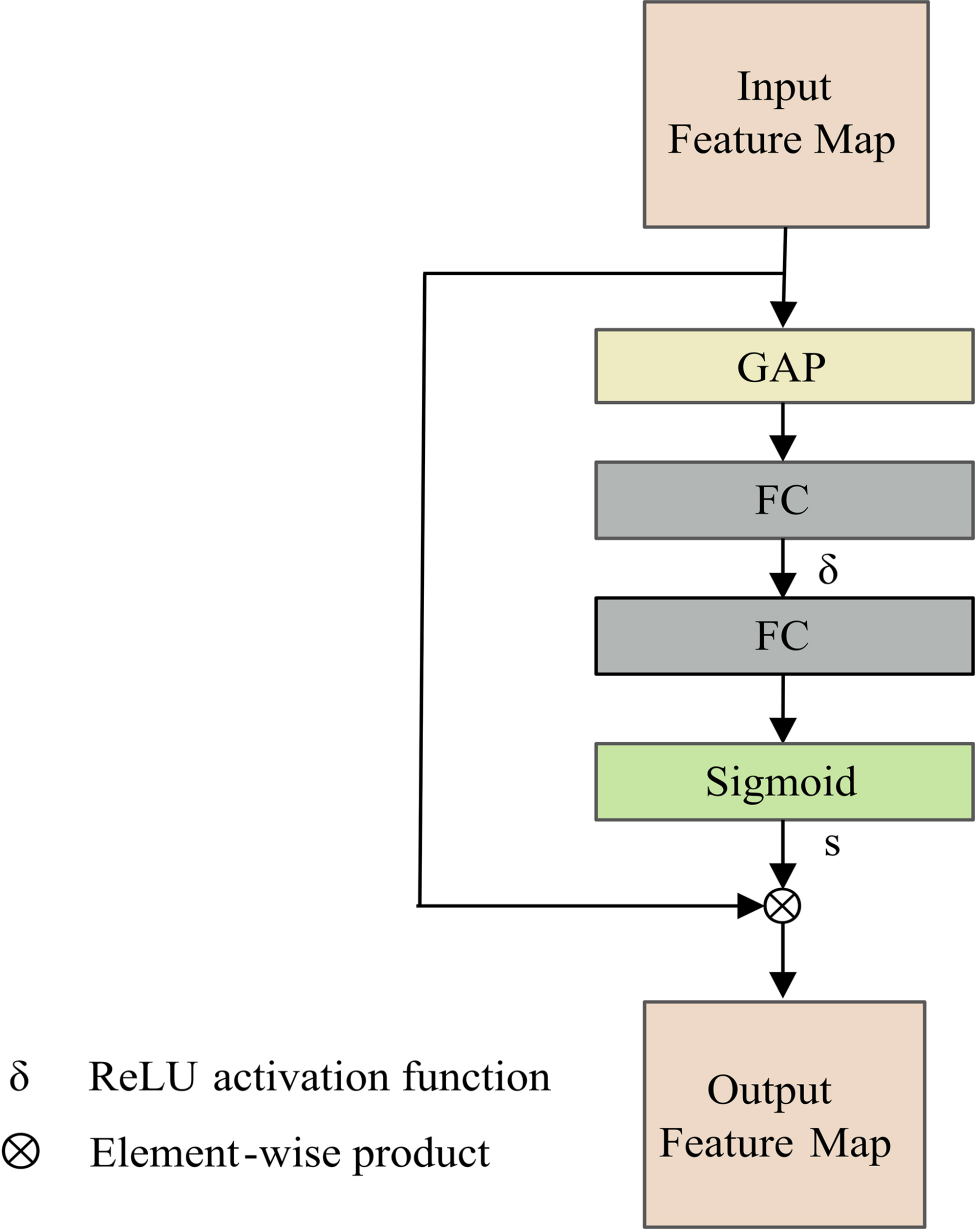
Squeeze and excitation block.

**Fig. (7):**
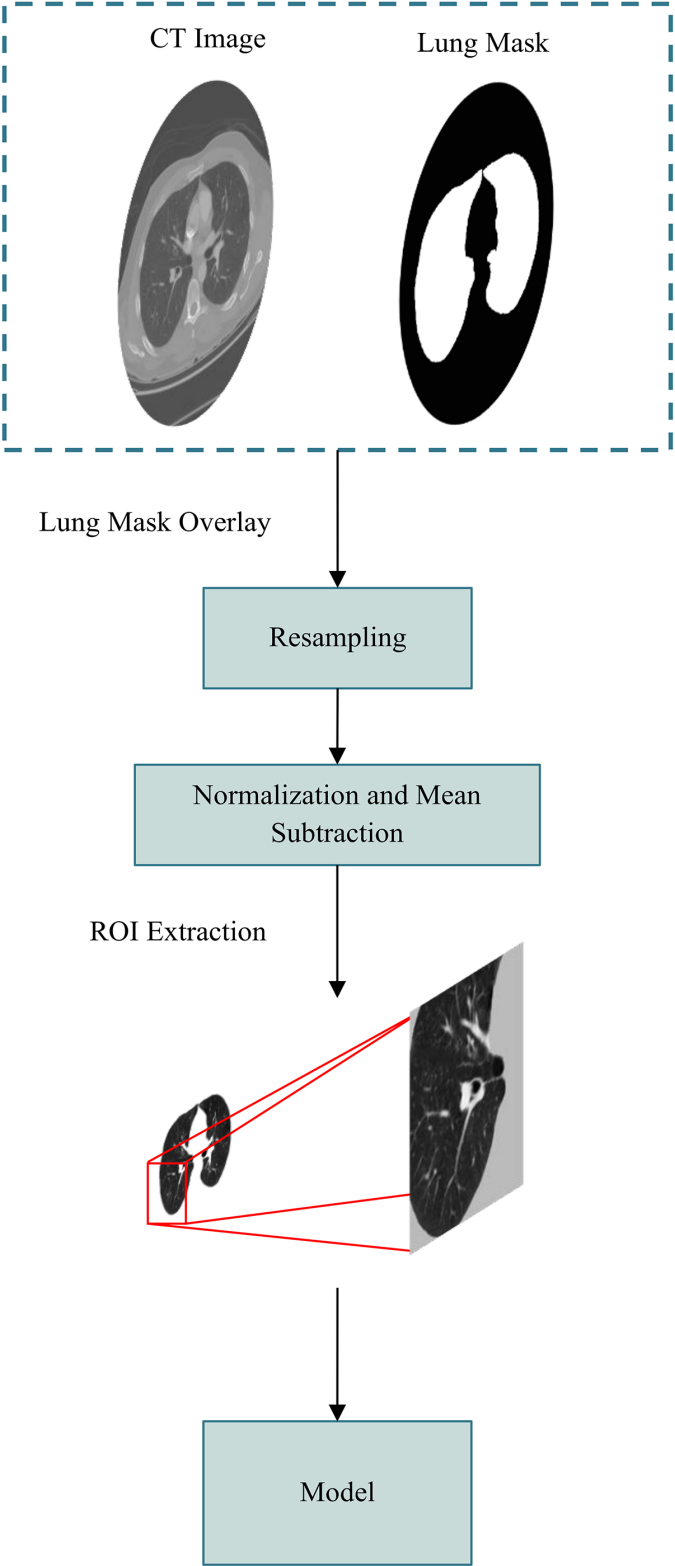
Overview of the preprocessing pipeline.

**Fig. (8):**
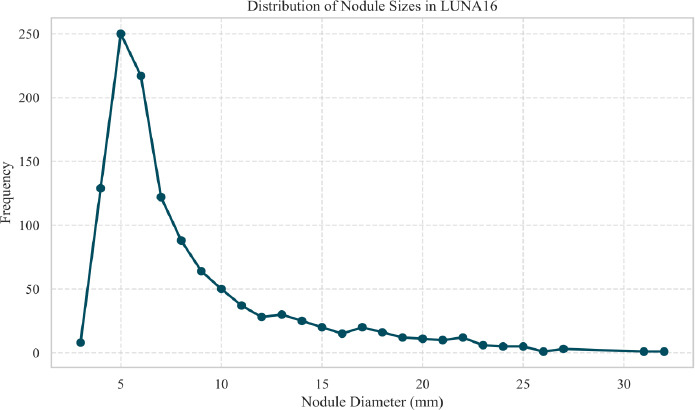
Distribution of nodule sizes in LUNA16 dataset.

**Fig. (9):**
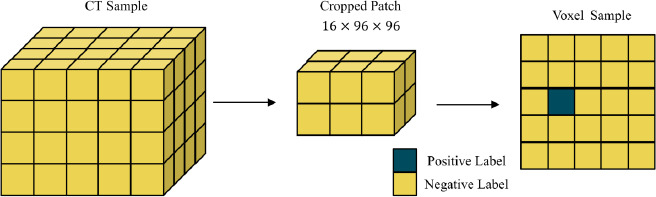
Illustration of patch extraction strategy. Each input patch is a 3D volume of size 16×96×96, where the majority of voxels are background (negative), and only a small portion corresponds to nodules (positive).

**Fig. (10):**
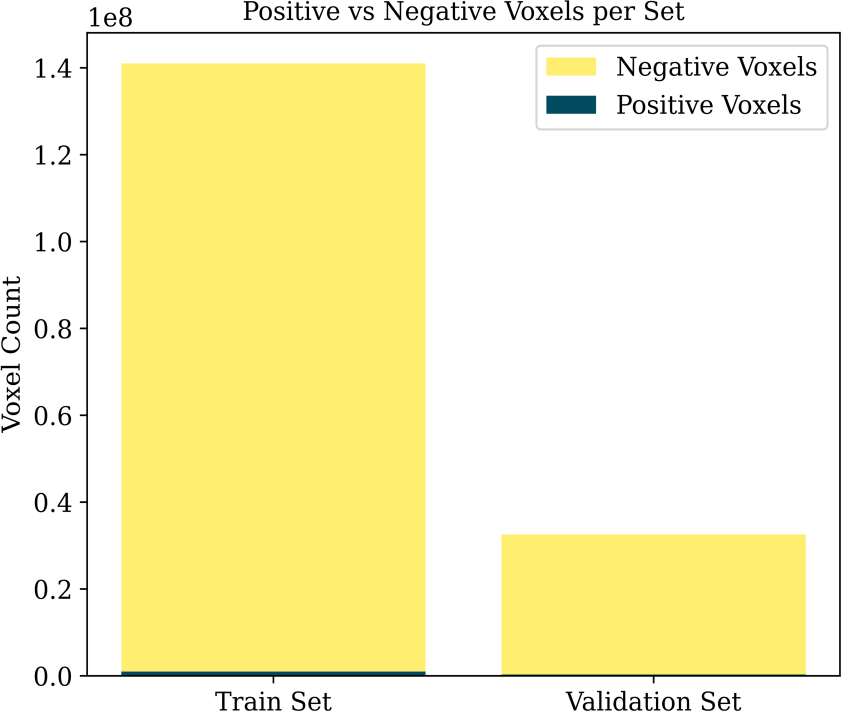
Voxel-level class distribution in the training and validation sets.

**Fig. (11):**
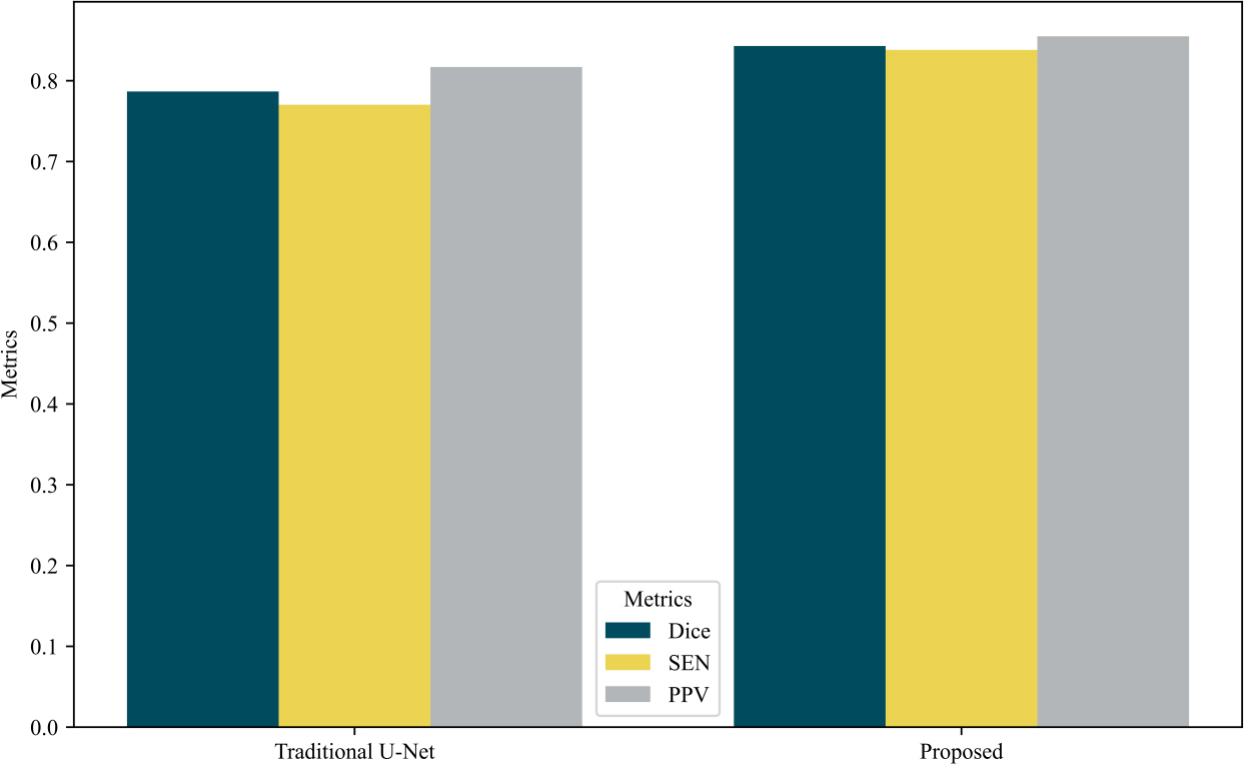
Comparison of segmentation performance between the traditional U-Net and the proposed model on the LUNA16 dataset using three evaluation metrics: Dice coefficient, Sensitivity (SEN), and Positive Predictive Value (PPV).

**Fig. (13):**
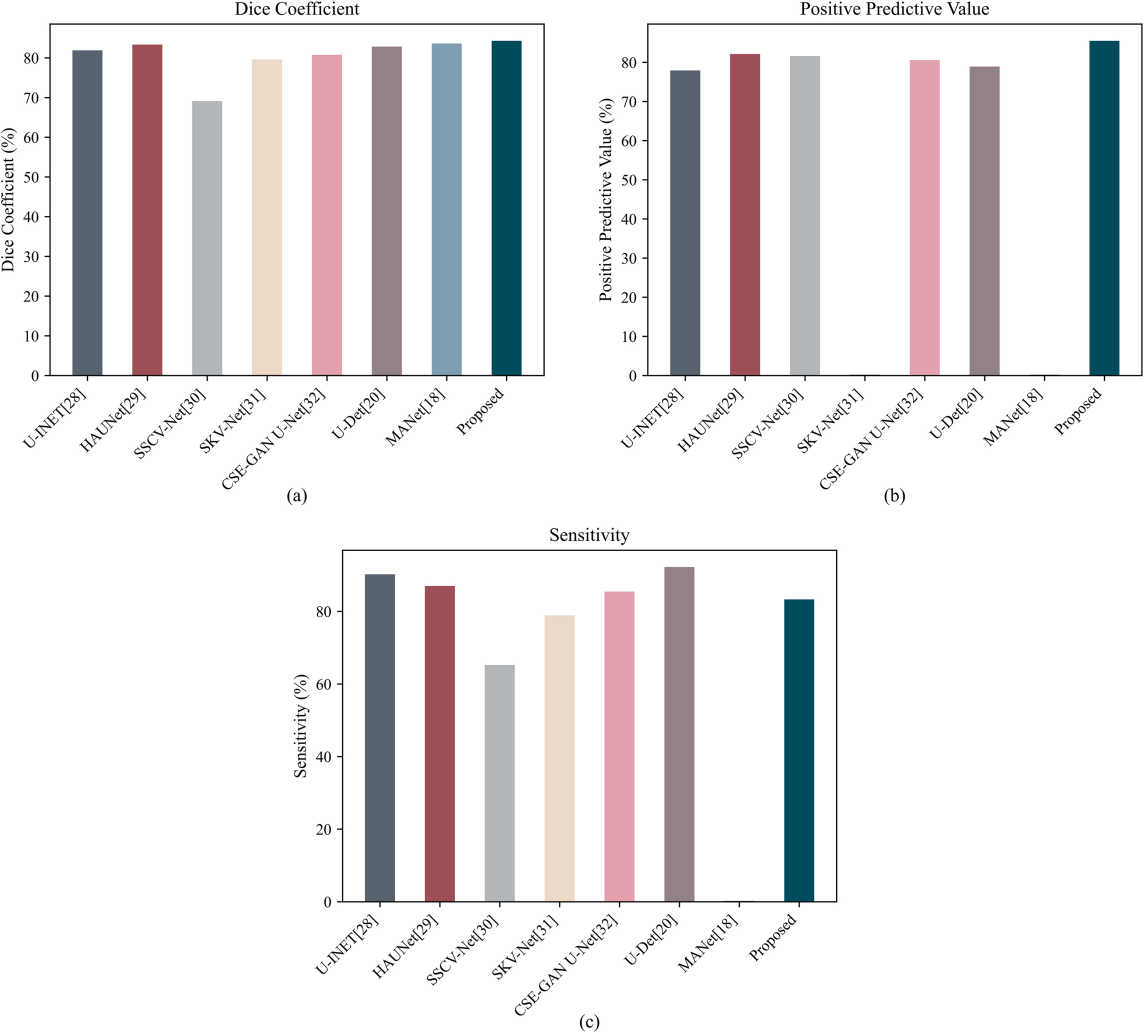
Comparison of segmentation performance across different enhanced models on the LUNA16 dataset. Subfigure (**a**) shows the Dice coefficient (DSC), (**b**) shows the Positive Predictive Value (PPV), and (**c**) shows the Sensitivity (SEN). The compared models include U-INET, HAU-Net, SSCV-Net, SKV-Net, CSE-GAN U-Net, U-Det, MANet, and the proposed model. Missing values are indicated with hatched bars.

**Fig. (14):**
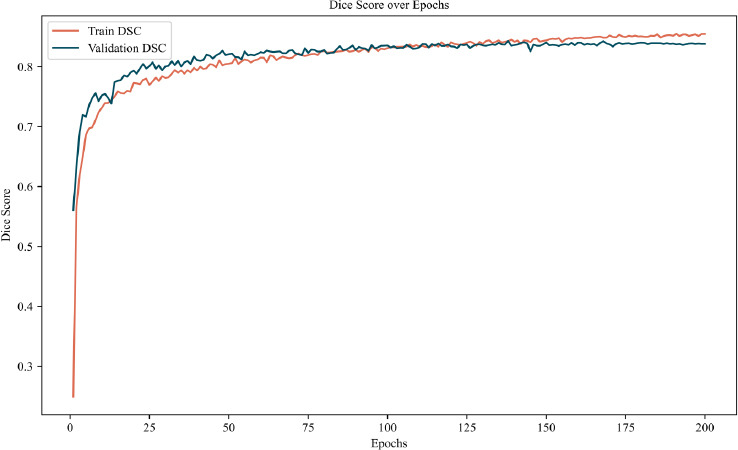
Evolution of Dice scores on the LUNA16 dataset across 200 epochs for both training and validation sets. The results highlight stable convergence and strong generalization of the proposed model.

**Fig. (15):**
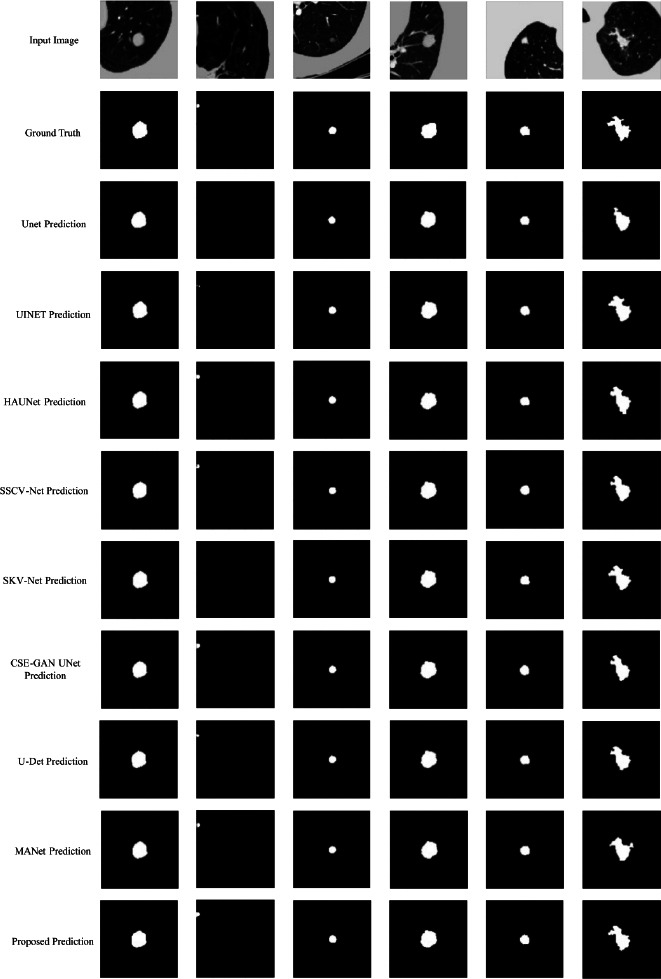
Visual comparison of lung nodule segmentation performance across six representative validation cases. Each column corresponds to one case selected to reflect common challenges such as low contrast, small size, irregular shape, and boundary ambiguity. From top to bottom, each row includes: the original CT slice, the ground truth mask, and the predicted segmentation masks from the following nine models in order, UNet, UINet, HAUNet, SSCV-Net, SKV-Net, CSE-GAN UNet, U-Det, MANet, and the proposed method.

**Table 1:** Model summary.

**Block**	**Layer**	**Output Shape**	**Details**
Encoder	DoubleConv3D	64 × 16 ×96 ×96	Two 3D convolutions with ReLU and BatchNorm, a residual connection
	Down3D	128 × 8 ×48 ×48	MaxPooling3D, DoubleConv3D, SpatialAttention3D, Dropout3D
	Down3D	256 × 4 ×24 ×24	MaxPooling3D, DoubleConv3D, SpatialAttention3D, Dropout3D
	Down3D	512 × 2 ×12 ×12	MaxPooling3D, DoubleConv3D, DilatedECA3D, Dropout3D
Decoder	Up3D	256 × 4 × 24 ×24	Trilinear Upsample, DoubleConv3D, CBAM3D, Dropout3D
	Up3D	128 × 8 × 48 × 48	Trilinear Upsample, DoubleConv3D, Dropout3D
	Up3D	64 × 16 × 96 × 96	Trilinear Upsample, DoubleConv3D, CBAM3D, Dropout3D
	OutputConv3D	1 × 16 ×96 ×96	Final 1 × 1 convolution to generate segmentation mask
Skip connections	SE Block 1	128 × 8 × 48 × 48	SE Block applied to the first skip connection
Skip connections	SE Block 2	512 × 2 × 12 × 12	SE Block applied to the third skip connection

**Table 2:** Details of datasets.

**Subset Index**	**Purpose**	**Sample Count**	**Label Count**
Subset 0–7	Training	963	963
Subset 8–9	Validation	223	223

**Table 3:** Performance comparison of optimizers.

**Optimizer**	**Dice**	**F1 Score**	**Average Epoch Time (s)**
Adam	0.8056	0.8111	95.45
AdamW	**0.8430**	**0.8465**	66.65
RAdam	0.8065	0.8109	**64.77**

**Table 4:** Comparison of different enhanced segmentation models based on the LUNA16 dataset.

**Model**	**Dataset Used**	**DSC (%)**	**PPV (%)**	**SEN (%)**
U-INET [28]	LUNA16	81.89	77.92	90.24
HAUNet [29]	LUNA16	83.34	82.12	87.01
SSCV-Net [30]	LUNA16	69.10	81.58	65.25
SKV-Net [31]	LUNA16	79.60	/	78.90
CSE-GAN U-Net [32]	LUNA16	80.74	80.56	85.46
U-Det [20]	LUNA16	82.82	78.92	**92.24**
MANet [18]	LUNA16	83.61	/	/
Proposed	LUNA16	**84.30**	**85.50**	83.32

## Data Availability

The data of current study are available from corresponding author, [A.A.K], on a reasonable request.

## LIST OF ABBREVIATIONS

CAD: Computer-Aided Diagnosis
CBAM: Convolutional Block Attention Module
CT: Computed Tomography
DSC: Dice Similarity Coefficient
PPV: Positive Predictive Value
ROI: Region of Interest
SA: Spatial Attention
SE: Squeeze-and-Excitation
SEN: Sensitivity

## References

[1] Zhou Q., Fan Y., Wang Y., Qiao Y., Wang G., Huang Y., Wang X., Wu N., Zhang G., Zheng X., Bu H. (2016). [China national guideline of classification, diagnosis and treatment for lung nodules (2016 version)].. Zhongguo Fei Ai Za Zhi.

[2] van Klaveren R.J., Oudkerk M., Prokop M., Scholten E.T., Nackaerts K., Vernhout R., van Iersel C.A., van den Bergh K.A.M., van ’t Westeinde S., van der Aalst C., Thunnissen E., Xu D.M., Wang Y., Zhao Y., Gietema H.A., de Hoop B.J., Groen H.J.M., de Bock G.H., van Ooijen P., Weenink C., Verschakelen J., Lammers J.W.J., Timens W., Willebrand D., Vink A., Mali W., de Koning H.J. (2009). Management of lung nodules detected by volume CT scanning.. N. Engl. J. Med..

[3] Aresta G., Jacobs C., Araújo T., Cunha A., Ramos I., van Ginneken B., Campilho A. (2019). iW-Net: An automatic and minimalistic interactive lung nodule segmentation deep network.. Sci. Rep..

[4] Lo S.C.B., Chan H.P., Lin J.S., Li H., Freedman M.T., Mun S.K. (1995). Artificial convolution neural network for medical image pattern recognition.. Neural Netw..

[5] Long J., Shelhamer E., Darrell T. Fully convolutional networks for semantic segmentation.. Proc IEEE Conf Comput Vis Pattern Recognit (CVPR).

[6] Ronneberger O., Fischer P., Brox T., Navab N, Hornegger J, Wells W (2015). U-Net: Convolutional networks for biomedical image segmentation.. Medical Image Computing and Computer-Assisted Intervention – MICCAI 2015.

[7] Zhou Z., Siddiquee M.M.R., Tajbakhsh N., Liang J. (2018). UNet++: A nested U-Net architecture for medical image segmentation.. Deep Learning in Medical Image Analysis and Multimodal Learning for Clinical Decision Support.

[8] Oktay O., Schlemper J., Folgoc L.L., Lee M., Heinrich M., Misawa K. (2018). Attention u-net: Learning where to look for the pancreas.. arXiv:1804.03999.

[9] Luo D., He Q., Ma M., Yan K., Liu D., Wang P. ECANodule: Accurate pulmonary nodule detection and segmentation with efficient channel attention.. Proceedings of the International Joint Conference on Neural Networks (IJCNN).

[10] Çiçek Ö., Abdulkadir A., Lienkamp S.S., Brox T., Ronneberger O., Ourselin S, Joskowicz L, Sabuncu M, Unal G, Wells W (2016). 3D U-Net: Learning dense volumetric segmentation from sparse annotation.. Medical Image Computing and Computer-Assisted Intervention – MICCAI 2016.

[11] Hu J., Shen L., Sun G., Albanie S., Wu E. Squeeze-and-excitation networks.. Proceedings of the IEEE Conference on Computer Vision and Pattern Recognition (CVPR).

[12] Woo S., Park J., Lee J-Y., Kweon I.S. CBAM: Convolutional block attention module.. Proceedings of the European Conference on Computer Vision (ECCV).

[13] Wang Q., Wu B., Zhu P., Li P., Zuo W., Hu Q. ECA-Net: Efficient Channel Attention for Deep Convolutional Neural Networks.. Proceedings of the IEEE/CVF Conference on Computer Vision and Pattern Recognition (CVPR).

[14] Hu B., Zhou P., Yu H., Dai Y., Wang M., Tan S., Sun Y. (2024). LeaNet: Lightweight U-shaped architecture for high-performance skin cancer image segmentation.. Comput. Biol. Med..

[15] Duan X., Sun Y., Wang J. (2023). ECA-UNet for coronary artery segmentation and three-dimensional reconstruction.. Signal Image Video Process..

[16] Ji Z., Zhao Z., Zeng X., Wang J., Zhao L., Zhang X., Ganchev I. (2023). ResDSda_U-Net: A novel u-net-based residual network for segmentation of pulmonary nodules in lung CT images.. IEEE Access.

[17] Sadremomtaz A., Zadnorouzi M. (2024). Improving the quality of pulmonary nodules segmentation using the new proposed U-Net neural network.. Intell. Based Med..

[18] Nguyen T.C., Nguyen T.P., Cao T., Dao T.T.P., Ho T.N., Nguyen T.V., Tran M.T. (2023). MANet: Multi-branch attention auxiliary learning for lung nodule detection and segmentation.. Comput. Methods Programs Biomed..

[19] Li X., Jiang A., Qiu Y., Li M., Zhang X., Yan S. (2023). TPFR-Net: U-shaped model for lung nodule segmentation based on transformer pooling and dual-attention feature reorganization.. Med. Biol. Eng. Comput..

[20] Annavarapu C.S.R., Parisapogu S.A.B., Keetha N.V., Donta P.K., Rajita G. (2023). A Bi-FPN-based encoder–decoder model for lung nodule image segmentation.. Diagnostics.

[21] Zeng Q., Zhou J., Tao J., Chen L., Niu X., Zhang Y. (2024). Multiscale global context network for semantic segmentation of high-resolution remote sensing images.. IEEE Trans. Geosci. Remote Sens..

[22] Milletari F., Navab N., Ahmadi S.A. V-Net: Fully convolutional neural networks for volumetric medical image segmentation.. Fourth International Conference on 3D Vision (3DV).

[23] Rezatofighi H., Tsoi N., Gwak J.Y., Sadeghian A., Reid I., Savarese S. Generalized Intersection over Union: A Metric and a Loss for Bounding Box Regression.. Proceedings of the IEEE/CVF Conference on Computer Vision and Pattern Recognition (CVPR).

[24] Kennedy J., Eberhart R. Particle swarm optimization.. Proceedings of the IEEE International Conference on Neural Networks (ICNN’95).

[25] Kingma D.P., Ba J. Adam: A method for stochastic optimization.. Proceedings of the 3rd International Conference on Learning Representations (ICLR).

[26] Loshchilov I., Hutter F. Decoupled weight decay regularization.. Proceedings of the International Conference on Learning Representations (ICLR).

[27] Liu L., Jiang H., He P. On the variance of the adaptive learning rate and beyond.. Proceedings of the 8th International Conference on Learning Representations (ICLR).

[28] Joshua E.S.N., Bhattacharyya D., Chakkravarthy M. (2023). Lung nodule semantic segmentation with bi-direction features using U-INET.. J Med Pharm Allied Sci.

[29] Zhou F., Luo F., Efio-Akolly K. HAUNet-3D: A novel hierarchical attention 3D unet for lung nodule segmentation.. Proceedings of the 2021 IEEE International Conference on Bioinformatics and Biomedicine (BIBM).

[30] Lin H., Zhang Y., Chen X., Wang H., Xia L. Research on pulmonary nodule segmentation algorithm based on improved V-Net.. 2022 IEEE 6th Advanced Information Technology, Electronic and Automation Control Conference (IAEAC ).

[31] Wang Z., Men J., Zhang F. (2023). Improved V-Net lung nodule segmentation method based on selective kernel.. Signal Image Video Process..

[32] Tyagi S., Talbar S.N. (2022). CSE-GAN: A 3D conditional generative adversarial network with concurrent squeeze-and-excitation blocks for lung nodule segmentation.. Comput. Biol. Med..

